# The genetic variation in drought resistance in eighteen perennial ryegrass varieties and the underlying adaptation mechanisms

**DOI:** 10.1186/s12870-023-04460-z

**Published:** 2023-09-26

**Authors:** Dan Wang, Yuting Zhang, Chunyan Chen, Ruixin Chen, Xuechun Bai, Zhiquan Qiang, Juanjuan Fu, Tao Qin

**Affiliations:** College of Grassland Agriculture, Northwest A&F University, Yangling, China

**Keywords:** Turfgrass, Perennial ryegrass, Drought stress, Genetic diversity, Molecular mechanism

## Abstract

**Background:**

Drought resistance is a complex characteristic closely related to the severity and duration of stress. Perennial ryegrass (*Lolium perenne* L.) has no distinct drought tolerance but often encounters drought stress seasonally. Although the response of perennial ryegrass to either extreme or moderate drought stress has been investigated, a comprehensive understanding of perennial ryegrass response to both conditions of drought stress is currently lacking.

**Results:**

In this study, we investigated the genetic variation in drought resistance in 18 perennial ryegrass varieties under both extreme and moderate drought conditions. The performance of these varieties exhibited obvious diversity, and the survival of perennial ryegrass under severe stress was not equal to good growth under moderate drought stress. ‘Sopin’, with superior performance under both stress conditions, was the best-performing variety. Transcriptome, physiological, and molecular analyses revealed that ‘Sopin’ adapted to drought stress through multiple sophisticated mechanisms. Under stress conditions, starch and sugar metabolic enzymes were highly expressed, while CslA was expressed at low levels in ‘Sopin’, promoting starch degradation and soluble sugar accumulation. The expression and activity of superoxide dismutase were significantly higher in ‘Sopin’, while the activity of peroxidase was lower, allowing for ‘Sopin’ to maintain a better balance between maintaining ROS signal transduction and alleviating oxidative damage. Furthermore, drought stress-related transcriptional and posttranscriptional regulatory mechanisms, including the upregulation of transcription factors, kinases, and E3 ubiquitin ligases, facilitate abscisic acid and stress signal transduction.

**Conclusion:**

Our study provides insights into the resistance of perennial ryegrass to both extreme and moderate droughts and the underlying mechanisms by which perennial ryegrass adapts to drought conditions.

**Supplementary Information:**

The online version contains supplementary material available at 10.1186/s12870-023-04460-z.

## Background

Drought is a major environmental stress affecting plant growth and development [1]. The impact of drought stress is expected to increase with climate change, especially the increase in the frequency, duration, and intensity of seasonal drought stress [2, 3]. As sessile organisms, plants have evolved specific and sophisticated mechanisms to cope with short- or long-term drought stresses [4]. Perennial ryegrass (*Lolium perenne* L.), native to Europe, Asia, and Northern Africa, is a cool-season perennial grass with a breeding history of more than 100 years [5, 6]. Due to its prominent lawn qualities, such as rapid establishment and attractive leafy appearance, perennial ryegrass is one of the most widely cultivated turfgrasses for golf courses, athletic fields, home lawns, and parks [7]. As a consequence of its wide distribution and perennial characteristics, perennial ryegrass often encounters and has to cope with a variety of abiotic stresses, including drought stress [1, 8]. Moreover, because perennial ryegrass has no distinct tolerance to drought, frequent watering is required throughout the whole lifecycle, and the established ryegrass sward is often challenged by drought stress [9, 10]. It is of great importance for sustainable lawns and landscapes to understand the response of perennial ryegrass to drought stress and integrate this knowledge into the process of breeding new cultivars with high drought resistance [8].

Genetic diversity is the heritable variation among intraspecific individuals [11]. The natural genetic diversity of plant species is an essential genetic resource for plant breeders to develop cultivars with desirable characteristics [12]. Most grass species usually have great diversity [11, 13]. Collecting natural or domesticated grass materials and assessing their genetic diversity are important steps for determining the determinant factors and breeding new varieties [13]. Perennial ryegrass is a self-incompatible diploid species with high genetic diversity within the population, which facilitates the introduction of drought resistance into perennial ryegrass by utilizing the genetic variation available in germplasm [10]. Moreover, the characterization and utilization of the genetic diversity of perennial ryegrass in response to drought stress are becoming increasingly important in view of climatic change with more frequent droughts and the demand for high-quality turfgrass in modern residential areas and sports facilities [11]. Plants can adapt to drought stress by stopping growth for survival under extreme conditions or continuing growth slowly under moderate stress. Increasing survival under severe drought does not indicate superior performance under mild drought and vice versa [14]. Thus, the drought resistance of plants is a complex and complicated characteristic closely related to the severity and duration of stress. Although studies on the response of perennial ryegrass to single extreme or moderate drought stress have been reported [7, 15–18], a comprehensive understanding of the genetic diversity of perennial ryegrass response to both extreme and moderate droughts remains to be further studied.

Even though the genetic diversity of stress resistance in perennial ryegrass germplasm facilitates the development of cultivars with high resistance, breeding new cultivars only by direct selection under stress conditions is time-consuming and often yields unpredictable results [15, 19]. It is widely accepted that identifying the genetic determinants and understanding the adaptation mechanisms of perennial ryegrass are of great significance for cultivating new varieties with strong stress resistance [19]. Understanding the genetic mechanisms and molecular markers is helpful for parental selection in breeding, and the identification of key determinant genes facilitates the improvement of perennial ryegrass by genetic engineering [6, 10]. Plant drought adaptation is also a quantitative trait involving multiple pathways. Numerous studies on the mechanisms and regulatory networks whereby *Arabidopsis thaliana* and annual crops adapt to drought stress have been published [4, 20, 21]. However, the mechanisms for perennial ryegrass may be different from those for annual plants due to its perennial feature. Most likely because of self‐incompatibility [22], incomplete coverage of reference genome information and poorly annotated transcriptome [5, 23], the adaptation mechanisms of perennial ryegrass are still poorly understood, even though a small number of drought-related genes of perennial ryegrass (e.g., *LpHUB1*, *LpP5CS*, and *LpSOD*) have been identified [24].

The objectives of this study were to investigate the natural variation in 18 perennial ryegrass varieties in response to both extreme and moderate droughts, explore the underlying adaptation mechanisms, and identify the potential genetic determinants. Such knowledge will provide insights into the genetic variation and potential mechanisms of perennial ryegrass response to drought stress.

## Results

### The survival and growth of different perennial ryegrass varieties under drought conditions

**Fig. 1:**
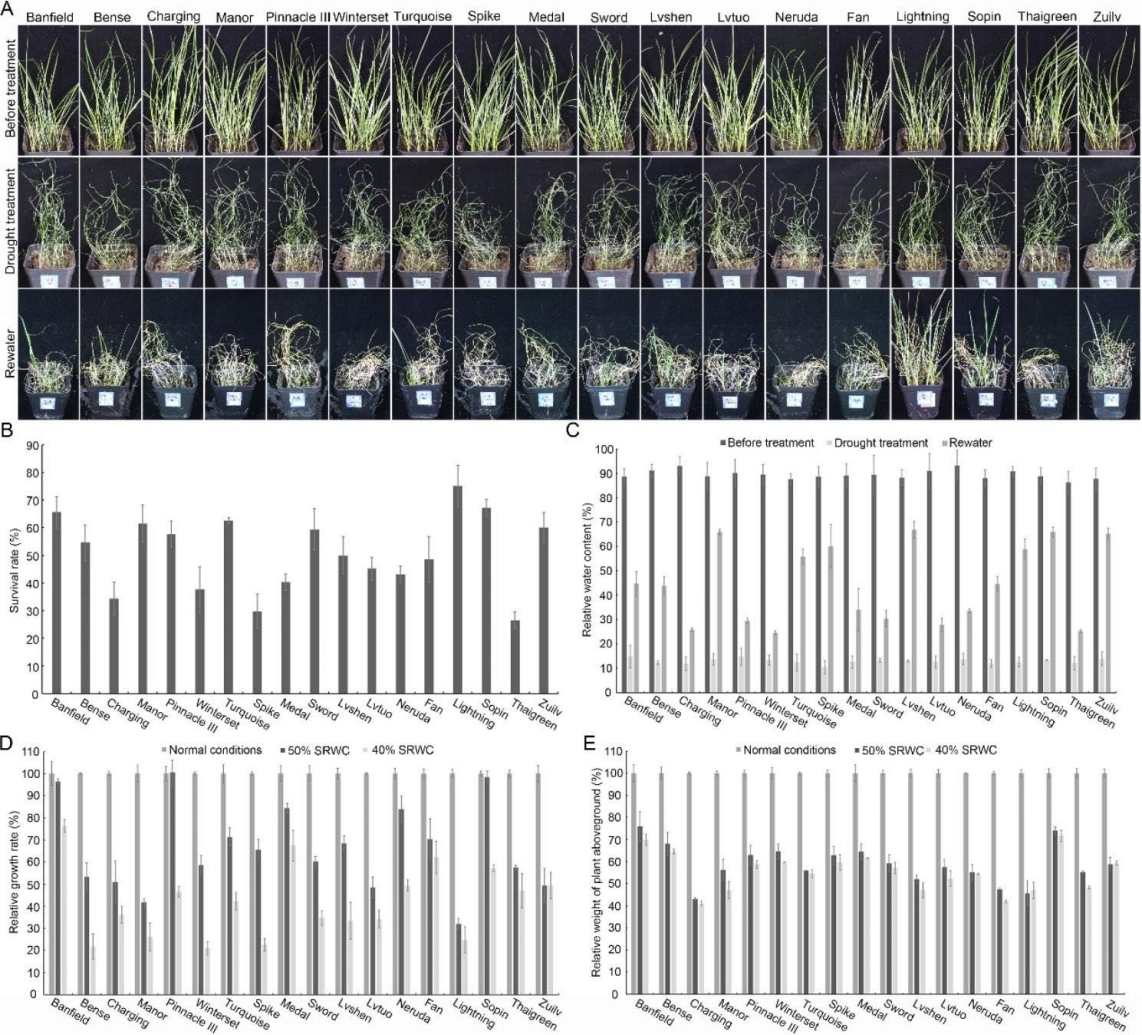
Seedling survival and growth of perennial ryegrass varieties under extreme or moderate drought stress. (**A**) Morphology of seedlings before and after extreme drought treatment. Photographs were taken before and after 30% soil relative water content (SRWC) treatment and 10 days after rewatering. (**B**) Seedling survival rate. At least 120 seedlings for each variety were scored. Data represent means ± SDs. (**C**) The relative water content of seedlings before and after 30% SRWC treatment and 10 days after rewatering. Data represent means ± SDs from four replicates. (**D**) The relative growth rate of seedlings growing under normal and moderate stress conditions. More than 100 seedlings for each variety under normal, 50% SRWC, or 40% SRWC conditions were scored. Data represent means ± SDs. (**E**) The relative aboveground biomass of each variety growing under normal and moderate stress conditions. Data represent means ± SDs from four replicates

To understand the genetic diversity of perennial ryegrass in response to droughts, 18 perennial ryegrass varieties commonly used for turf establishment in China were used as research materials. To avoid the potential confounding effects related to light, temperature, or soil nutrient deficiency in the field, we evaluated the genetic variation of perennial ryegrass varieties in a growth room with fixed growth conditions and commercialized nutrient soil. First, the genetic variation in these varieties under extreme drought conditions was investigated. According to a previous report that a soil relative water content (SRWC) less than 30% represents extreme drought stress for perennial rhizome grass and perennial bunchgrass [25], 17-day-old well-watered seedlings of each variety were subjected to stress of 30% SRWC for 2 weeks. The results showed that all leaves of seedlings became totally withered, but the leaves of ‘Lightning’, ‘Sopin’, and ‘Banfield’ withered to a lesser extent, and seedling bases were not parched (Fig. 1A). Ten days after rewatering, some leaves of ‘Lightning’, ‘Sopin’, and ‘Zuilv’ became green again, while almost all dried leaves of the other varieties were dead (Fig. 1A). ‘Lightning’, ‘Sopin’, and ‘Banfield’ were the most tolerant varieties, with survival rates greater than 65%. In contrast, ‘Thaigreen’, ‘Spike’, and ‘Charging’ were the most susceptible varieties, with survival rates of less than 35% (Fig. 1B). We also treated seedlings of each variety with 20% SRWC for 10 days. Similar to the 30% SRWC treatment, the leaves of all seedlings withered after the stress treatment (Fig. S1A). However, ‘Bense’ and ‘Zuilv’ were more susceptible to more severe but shorter drought stress, while ‘Medal’ and ‘Lvshen’ varieties were more tolerant to these stress conditions. The survival rates of ‘Bense’ and ‘Zuilv’ decreased, while those of ‘Medal’ and ‘Lvshen’ increased significantly (Fig. S1A). The most tolerant varieties were ‘Lvshen’, ‘Medal’, and ‘Sopin’, with survival rates higher than 90%. The most susceptible varieties were ‘Bense’, ‘Winterset’, and ‘Zuilv’, with survival rates of less than 30% (Fig. S1B). We also evaluated the relative water content of each variety before and after stress treatment to fully evaluate the tolerance of these varieties to extreme drought stress. The relative water contents of all varieties were nearly 90% before drought treatment. However, the contents decreased to less than 15% when seedlings were subjected to 30% SRWC for 2 weeks (Fig. 1C). After rewatering, ‘Lvshen’, ‘Sopin’, and ‘Manor’ were the varieties with the highest relative water contents, which were 66.8%, 66.0%, and 65.9%, respectively. In contrast, ‘Winterset’, ‘Thaigreen’, and ‘Charging’, with relative water contents of 24.6%, 25.3%, and 25.8%, respectively, were the most intolerant varieties (Fig. 1C). These data demonstrate that ‘Sopin’ performs well in all analyses and is one of the most resistant varieties to extreme drought stress.

Due to irrigation, stochastic moderate drought rather than extreme drought is more common in lawn construction and maintenance. The investigation of the genetic variation in perennial ryegrass varieties under moderate drought stress is also important. Seventeen-day-old well-watered seedlings of each variety were subjected to 50% or 40% SRWC for 2 weeks. Compared with seedlings under normal conditions, the height of seedlings growing under stress conditions decreased significantly, and the degree of stunted growth was in line with the severity of stress conditions (Fig. S2). The varieties with the least growth inhibition under 50% SRWC were ‘Pinnacle III’, ‘Sopin’, and ‘Banfield’. Their growth seemed unaffected by stress. In contrast, ‘Lightning’, ‘Manor’, and ‘Lvtuo’ were the most susceptible varieties, with relative growth rates of 32.0%, 41.8%, and 48.5%, respectively (Fig. 1D). Under 40% SRWC, ‘Banfield’, ‘Medal’, and ‘Fan’ with relative growth rates over 62% were the best-performing varieties, while ‘Winterset’, ‘Bense’, and ‘Spike’ with relative growth rates lower than 23% were the poorest-growing varieties (Fig. 1D). The aboveground biomass of each variety also decreased significantly under moderate stress conditions, but there was little difference between the relative weights of the same variety under 50% and 40% SRWC (Fig. 1E). The relative weights of ‘Sopin’, ‘Banfield’, and ‘Bense’ were the highest, more than 65%, while those of ‘Charging’, ‘Fan’, and ‘Lightning’ were less than 47% (Fig. 1E). These data demonstrate that ‘Banfield’ is one of the best-performing varieties under moderate drought conditions.

### PCA of phenotypic and physiological traits associated with drought resistance in 18 perennial ryegrass varieties

**Fig. 2:**
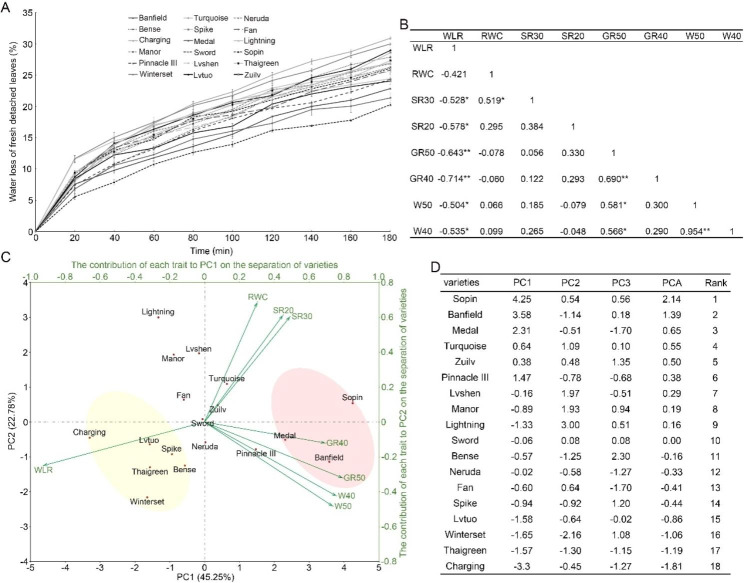
Principal component analysis of 18 perennial ryegrass cultivars based on data of phenotypic and physiological traits. (**A**) Transpiration water loss rates of detached leaves at the indicated time points after detachment. The data presented in this plot represent means ± SDs from three replicates. (**B**) Pearson correlation coefficient analysis among trait values. WLR, water loss rate of detached leaves; RWC, the ratio of relative water content after rewatering to that before stress treatment; SR30, survival rate under 30% SRWC; SR20, survival rate under 20% SRWC; GR50, relative growth rate under 50% SRWC; GR40, relative growth rate under 40% SRWC; W50, relative aboveground weight under 50% SRWC; W40, relative aboveground weight under 40% SRWC. * and ** indicate significance at *P* < 0.05 and *P* < 0.01, respectively. (**C**) PCA biplot of 8 traits of 18 perennial ryegrass varieties. Arrows represent phenotypic and physiological traits with various lengths based on their impact on the separation of perennial ryegrass varieties. The most resistant varieties based on PC1 and PC2 are marked with a pink background, and varieties with the lowest stress resistance are marked with a yellow background. (**D**) The three major components (PC1, PC2, and PC3) and PCA ranking of each variety

We also measured the water loss rate of detached leaves, which usually represents the water loss rate by transpiration [26]. As shown in Fig. 2A, the water loss rates of ‘Sopin’, ‘Medal’, and ‘Banfield’ were much slower than those of the other varieties. Their leaves lost 20.3%, 21.4%, and 22.9% of their fresh weight at 180 min after detachment. In contrast, ‘Charging’ and ‘Winterset’ lost more than 30.1% of their fresh weight (Fig. 2A).

To evaluate the correlation relationship between these phenotypic and physiological traits and gain insight into the overall drought tolerance of the 18 perennial ryegrass varieties, principal component analysis (PCA) was performed with traits of water loss rate of detached leaves (WLR), the ratio of relative water content after rewatering with that before stress treatment (RWC), survival rates under 30% and 20% SRWC conditions (SR30 and SR20), relative growth rates under 50% and 40% SRWC conditions (GR50 and GR40), and relative aboveground weights under 50% and 40% SRWC (W50 and W40) (Table S1). First, the relationship between these traits was assessed by Pearson correlation analysis. The results demonstrated that WLR had larger correlation coefficients with other traits, indicating that water loss by transpiration was associated with the overall tolerance of perennial ryegrass to both extreme and moderate drought stresses. However, SR30 and SR20 had low correlation coefficients with GR50, GR40, W50, or W40, which was consistent with the view that the survival under extreme drought conditions is irrelevant to the performance under moderate drought stress [14] (Fig. 2B). Kaiser-Meyer-Olkin (KMO) and Bartlett’s test on trait values showed that the KMO measure of sampling adequacy was > 0.5, and the significance of Bartlett’s test was < 0.05, indicating that these traits can be used for PCA (Fig. S3A). Based on these traits, 8 principal components (PC1- PC8) were identified, among which the eigenvalues of the first three principal components were > 1 (Fig. S3B). The major principal components PC1 and PC2 explained 45.3% and 22.6% of the variance, respectively, and the sum of the first three principal components explained 84.4% of the variance among the 18 perennial ryegrass varieties (Fig. S3B).

Based on the component matrix^α^ and values of each trait (Fig. S3C and Table S1), the following formulas for ranking the relative drought tolerance of the 18 perennial ryegrass varieties were developed: PC1 = (− 0.487) × ZWLR + 0.160 × ZRWC + 0.258 × ZSR30 + 0.237 × ZSR20 + 0.417 × ZGR50 + 0.365 × ZGR40 + 0.387 × ZW50 + 0.398 × ZW40; PC2 = (− 0.185) × ZWLR + 0.511 × ZRWC + 0.449 × ZSR30 + 0.455 × ZSR20 + (− 0.238) × ZGR50 + (− 0.089) × ZGR40 + (− 0.357) × ZW50 + (− 0.314) × ZW40; PC3 = 0.103 × ZWLR + 0.379 × ZRWC + 0.305 × ZSR30 + (− 0.373) × ZSR20 + (− 0.300) × ZGR50 + (− 0.478) × ZGR40 + 0.369 × ZW50 + 0.394 × ZW40; and PCA = 0.453 × PC1 + 0.226 × PC2 + 0.165 × PC3. ZWLR, ZRWC, ZSR30, ZSR20, ZGR50, ZGR40, ZW50, and ZW40 in these formulas represent standardized values of WLR, RWC, SR30, SR20, GR50, GR40, W50, and W40, respectively (Table S1). Among these traits, WLR was negatively associated with the overall drought tolerance of perennial ryegrass, while RWC, SR20, and SR30 were positively correlated with drought tolerance. However, the contribution of GR50, GR40, W50, and W40 to stress tolerance was multifaceted, with both positive and negative effects, indicating that the relationship between rapid growth under stress and drought resistance was intricate (Fig. 2C). WLR and GR50 contributed more than other traits to the separation of the 18 ryegrass varieties, which were clustered into three groups (Fig. 2C). The first group consisted of three varieties: ‘Sopin’, ‘Banfield’, and ‘Medal’. These varieties were negatively correlated with WLR but positively correlated with other traits, thereby with PCA rank values at the top of all varieties. The second group was represented by ‘Charging’, ‘Winterset’, ‘Thaigreen’, ‘Lvtuo’, ‘Spike’, and ‘Bense’. These varieties were positively correlated with WLR and negatively correlated with other traits, thus having the lowest PCA rank values. The remaining varieties were clustered into the third group, and their rank values were at the middle levels of all varieties (Fig. 2C and D). ‘Sopin’, with superior performance under extreme or moderate stress conditions and the highest PCA rank value, had the highest relative drought tolerance among these varieties. ‘Charging’, which had the worst overall performance under stress conditions and the lowest rank value, was the most susceptible variety (Fig. 2D).

### RNA-seq analysis of the transcriptomic difference between the tolerant and susceptible varieties

**Fig. 3:**
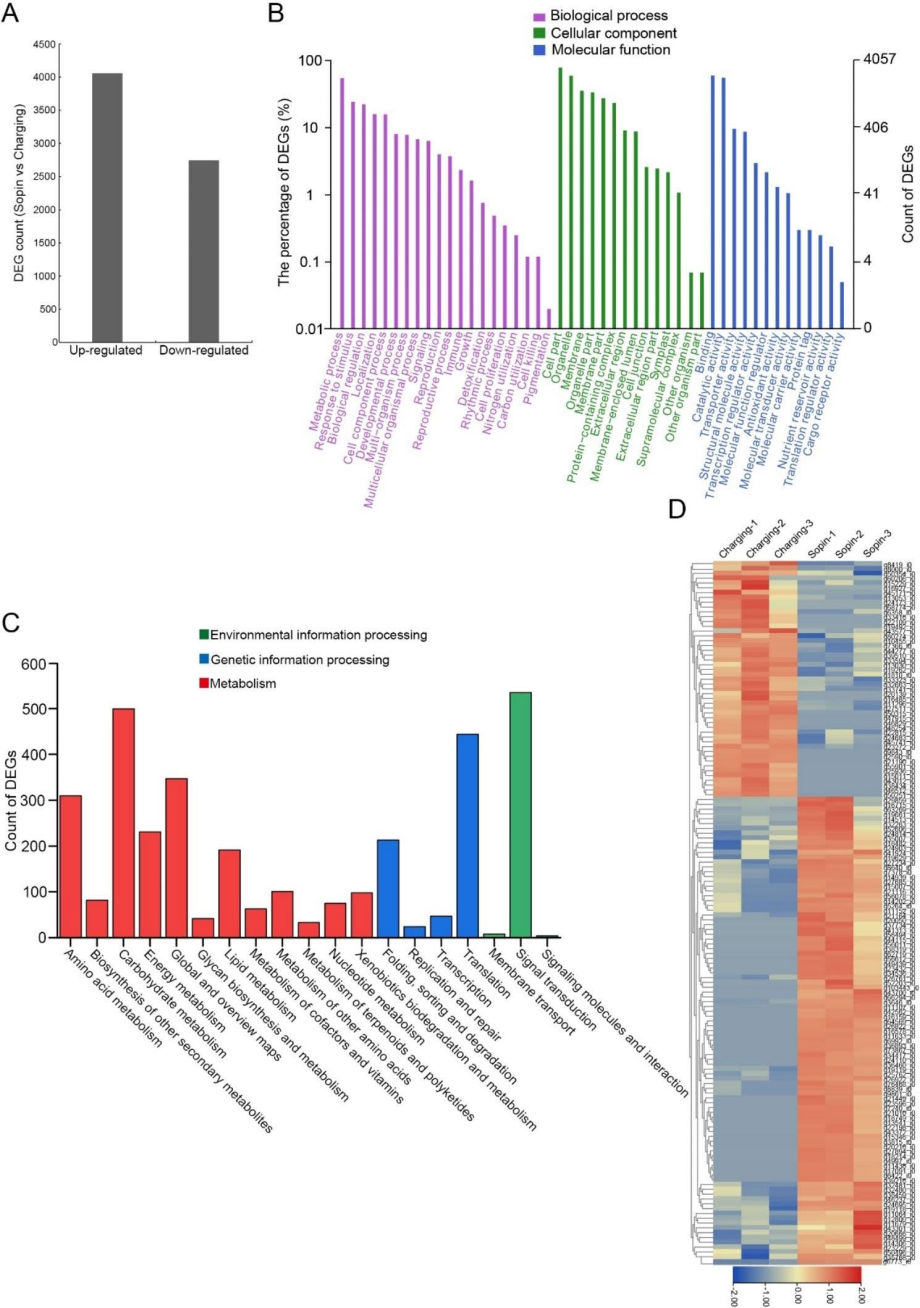
Transcriptome analysis of ‘Sopin’ and ‘Charging’ under dehydration treatment. (**A**) The number of DEGs upregulated or downregulated in ‘Sopin’. (**B**) GO terms of DEGs. The abscissa is the GO classifications. The left and right ordinates are the percentage or count of DEGs for each GO term. (**C**) The KEGG pathways for DEGs. (**D**) Heatmap diagrams showing the expression pattern of DEGs that are potentially related to drought resistance. Red and blue colors indicate a log2-fold change in expression upregulation or downregulation, respectively

Because the adjustment of plant development and metabolism is determined by genes, identifying the differentially expressed genes (DEGs) under drought stress may help to elucidate the potential molecular mechanisms [10]. To investigate the mechanisms of perennial ryegrass adaptation to drought stress, transcriptome analysis was performed using 7-day-old seedlings of ‘Sopin’ and ‘Charging’ treated by dehydration, which is often used to mimic drought stress [26]. The results showed that there were 6,803 genes with significant differences, of which 4,058 genes were upregulated and 2,745 genes were downregulated in ‘Sopin’ (Fig. 3A and Table S2).

To identify the major functional categories of DEGs, Gene Ontology (GO) and Kyoto Encyclopedia of Genes and Genomes (KEGG) enrichment analyses were carried out. The results showed that these DEGs could be categorized into 47 GO terms. Nine hundred eighty-six DEGs were enriched in ‘Response to stimulus’, a stress-related GO term and the second largest class in the biological process category. Fifty-three DEGs were enriched in the term ‘Antioxidant activity’, which is particularly related to oxidative stress (Fig. 3B). KEGG results showed that these DEGs were enriched in 19 pathways. The 5 most significantly enriched pathways were involved in ‘signal transduction’, ‘carbohydrate metabolism’, ‘translation’, ‘global and overview maps’, and ‘biosynthesis of amino acids’, implying that these DEGs might be relevant for drought tolerance by modulating signal transduction, protein regulation and metabolism of carbohydrates and amino acids (Fig. 3C). Based on the annotation of DEGs, we identified 146 DEGs that were potentially related to drought resistance, including 97 upregulated genes and 49 downregulated genes (Fig. 3D). These genes were mainly involved in stress signal transduction by functioning as transcription factors, kinases, or E3 ubiquitin-protein ligases; involved in osmolyte metabolism by functioning as key enzymes or regulators; and involved in detoxication of oxidative stress by functioning as key antioxidant enzymes (Table S3).

### The different osmolyte metabolism strategies of ‘Sopin’ and ‘Charging’

**Fig. 4:**
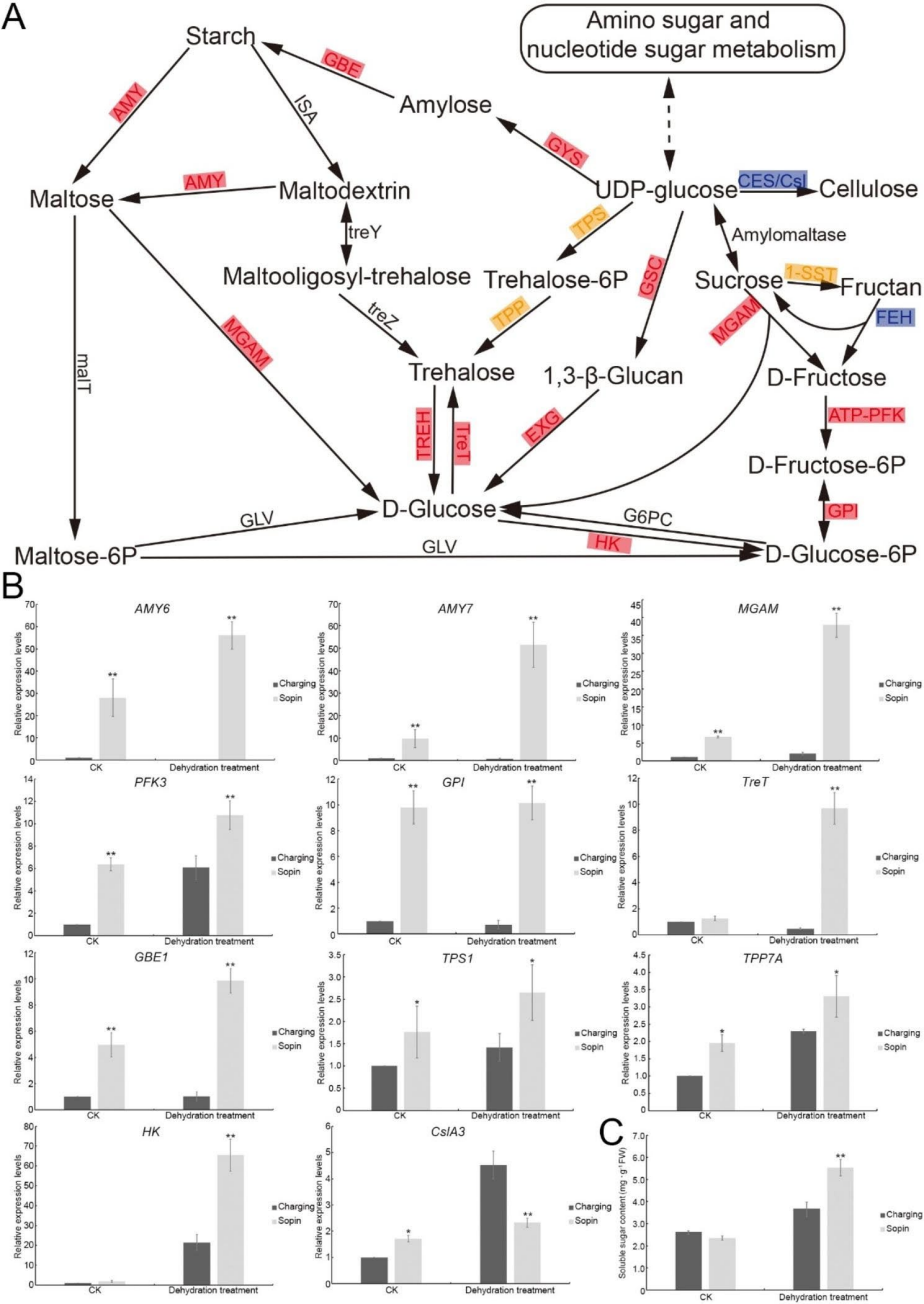
The metabolism of starch and sucrose in ‘Sopin’ and ‘Charging’ under dehydration treatment. (**A**) The starch and sucrose metabolic pathway based on the KEGG database (ko00500). In transcriptome analysis, enzymes upregulated in ‘Sopin’ are labeled with red boxes, enzymes downregulated in ‘Sopin’ are labeled with blue boxes, and the yellow box labeled enzymes are coded by genes that were both upregulated and downregulated in ‘Sopin’. (**B**) Relative expression levels of selected genes involved in starch and sucrose metabolism by RT-qPCR. *, *P* < 0.05 and **, *P* < 0.01 by Student’s *t*-test compared with gene expression levels in ‘Charging’. Data represent means ± SDs from three replicates. (**C**) The soluble sugar content. **, *P* < 0.01 by Student’s *t*-test compared with the content in ‘Charging’. Data represent means ± SDs from four replicates

In our transcriptome analysis, 499 DEGs were involved in ‘Carbohydrate metabolism’ (Fig. 3C), among which 90 DEGs were particularly involved in starch and sucrose metabolism (Fig. S4). In addition, among these potential drought stress-related DEGs, 43 genes were involved in soluble sugar metabolism, and 30 of them participated in starch or sucrose metabolism (Table S3). The metabolism of starch and sucrose plays a crucial role in coping with drought stress [27]. We focused on DEGs that specifically regulate starch and sucrose metabolism. The RNA-seq results showed that even though genes encoding trehalose-6-phosphate synthase (TPS), trehalose-6-phosphate phosphatase (TPP), and sucrose:sucrose-1-fructosyl transferase (1-SST), the key enzymes involved in the synthesis of trehalose or fructans [28, 29], were both upregulated and downregulated in ‘Sopin’, the expression levels of most other key enzymes, such as amylase (AMY), which modulates the degradation of starch under stress conditions [27] and hexokinase (HK), which confers plant drought resistance by modulating glucose homeostasis and glucose signaling [30], were upregulated. In contrast, cellulose synthase (CES) and cellulose synthase-like (Csl) which catalyze the biosynthesis of insoluble cellulose and hemicellulose in plants [31], as well as fructan exohydrolase (FEH) that catalyzes the breakdown of fructans [32], were downregulated in ‘Sopin’ (Fig. 4A and Table S3). To further confirm this conclusion, the expression levels of representative genes were evaluated by RT-qPCR. As shown in Fig. 4B, the expression levels of *AMY6*, *AMY7*, *maltase-glucoamylase* (*MGAM*), *phosphofructokinase3* (*PFK3*), *glucose-6-phosphate isomerase* (*GPI*), *trehalose glycosyltransferase synthase* (*TreT*), *glycogen-branching enzyme 1* (*GBE1*), *TPS1*, *TPP7A*, and *HK* were significantly higher in ‘Sopin’ than in ‘Charging’ under dehydration treatment. However, the expression level of *CslA3* was lower under stress conditions (Fig. 4B). Fructans are the major reserve of soluble carbohydrates in temperate grasses. Apart from their role as storage carbohydrates, fructans are believed to confer drought tolerance [33–36]. The transcription level of *1-SSTa* was slightly higher in ‘Sopin’ than in ‘Charging’, while the levels of *1-SSTb* and *1-SSTc* were lower in ‘Sopin’ under dehydration conditions (Fig. S5), which is consistent with the notion that fructosyltransferases (FTs) are regulated by transcriptional and posttranslational mechanisms, as well as the fact that the fructan concentration is not always in line with the expression levels of FTs [37, 38]. Intriguingly, the expression level of *FEH* was lower in ‘Sopin’ under dehydration conditions (Fig. S5). These data imply that more soluble sugar, e.g. glucose, sucrose, trehalose, and fructan, accumulated in ‘Sopin’ under stress conditions. The results of the analysis of soluble sugar content showed that there was no significant difference between the two varieties under normal conditions. The contents in ‘Sopin’ and ‘Charging’ were 2.4 and 2.6 mg/g fresh weight, respectively. After dehydration treatment, the content of soluble sugar in ‘Sopin’ increased dramatically, which was 1.5-fold that in ‘Charging’ (Fig. 4C), demonstrating that the metabolism of soluble sugar should be one of the mechanisms by which ‘Sopin’ adapts to drought stress.

Intriguingly, among the 310 DEGs involved in ‘amino acid metabolism’ (Fig. 3C), 126 DEGs specifically participated in the biosynthesis of amino acids, including proline (Fig. S6). Among these genes related to the biosynthesis of proline, genes coding Δ1-pyrroline-5-carboxylate synthase (P5CS) and glutamate-5-kinase (G5K), which catalyze the controlling first step of proline biosynthesis from glutamate, respectively [39, 40], were downregulated in ‘Sopin’. Whereas other enzymes, i.e., glutamate-pyruvate transferase (GPT), aldehyde dehydrogenase (ALDH), N-acetylglutamate kinase (AGK), N-acetylornithine aminotransferase (ACOAT), and ornithine-delta-aminotransferase (OAT), were upregulated (Fig. S7A). The results of RT-qPCR showed that the expression levels of *GPT*, *AGK*, *ACOAT*, *OAT2*, and pyrroline-5-carboxylate reductase 1 (*P5CR1*) in ‘Sopin’ were at least 2.5-fold of those in ‘Charging’. However, the expression of *P5CS* was much lower in ‘Sopin’ under dehydration treatment (Fig. S7B). Measuring the proline content, the results showed that the proline level in ‘Sopin’ was significantly lower than that in ‘Charging’ (Fig. S7C). These results demonstrate that ‘Sopin’ and ‘Charging’ probably adopt different strategies to alleviate osmotic stress. Under stress conditions, more soluble sugar accumulated in ‘Sopin’ whereas more proline accumulated in ‘Charging’.

### The maintenance of the balance between ROS-modulated signal transduction and oxidative damage

**Fig. 5:**
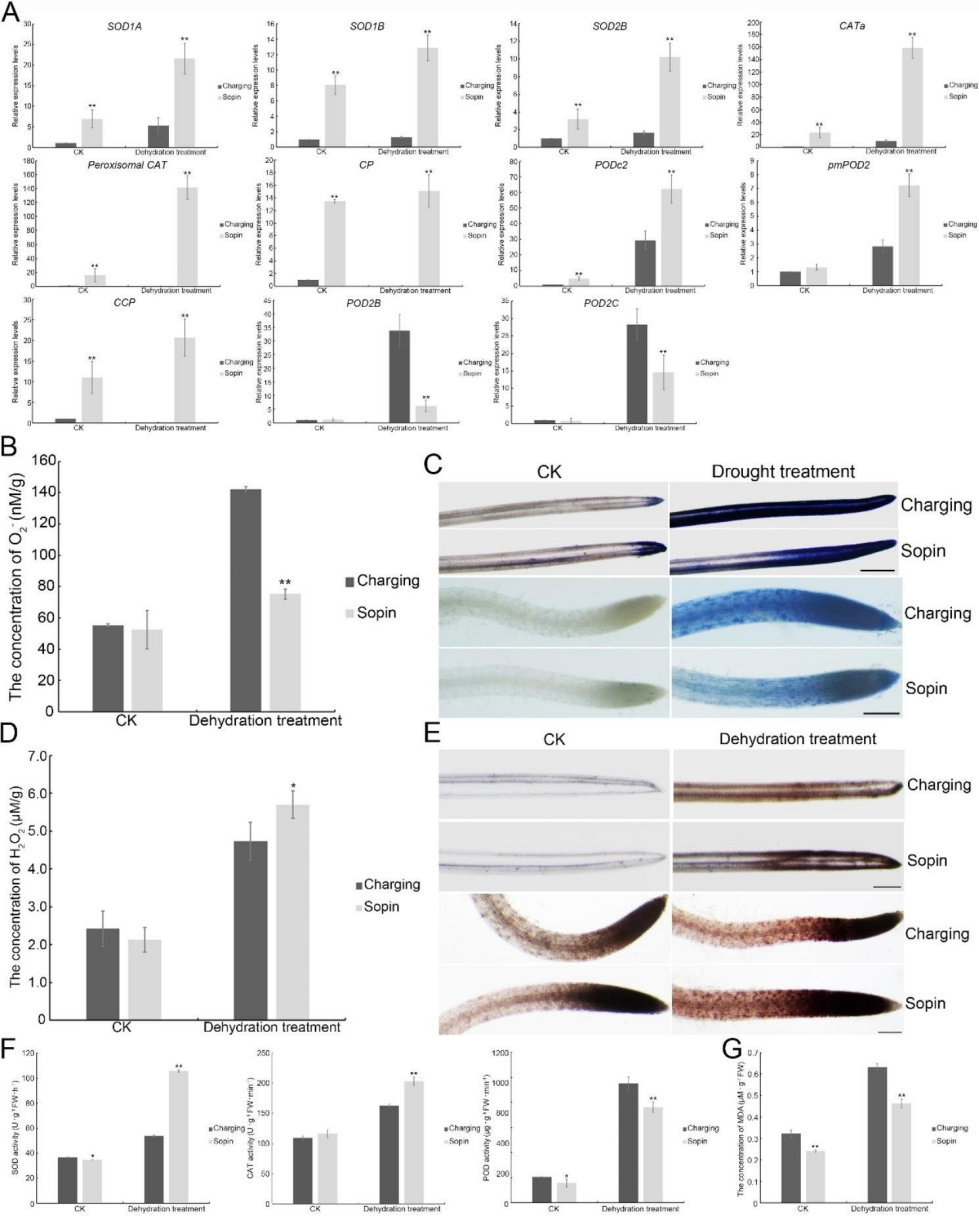
ROS homeostasis in ‘Sopin’ and ‘Charging’ under dehydration treatment. (**A**) Relative expression levels of representative antioxidant genes by RT-qPCR. **, *P* < 0.01 by Student’s *t*-test compared with gene expression levels in ‘Charging’. Data represent means ± SDs from three replicates. (**B**) The concentration of O_2_^−^. **, *P* < 0.01 by Student’s *t*-test compared with the concentration of O_2_^−^ in ‘Charging’. Data represent means ± SDs from four replicates. (**C**) The content of O_2_^−^ in the leaves and roots stained by NBT. Bars in leaf and root images represent 1 mm and 200 μm, respectively. (**D**) The concentration of H_2_O_2_. *, *P* < 0.05 by Student’s *t*-test compared with the concentration of H_2_O_2_ in ‘Charging’. Data represent means ± SDs from four replicates. (**E**) The content of H_2_O_2_ in the leaves and roots stained by DAB. Bars in leaf and root images represent 500 μm and 200 μm, respectively. (**F**) The activities of SOD, CAT, and POD. *, *P* < 0.05 and **, *P* < 0.01 by Student’s *t*-test compared with the activities in ‘Charging’. Data represent means ± SDs from three replicates. (**G**) The concentration of MDA. **, *P* < 0.01 by Student’s *t*-test compared with the concentration of MDA in ‘Charging’. Data represent means ± SDs from four replicates

Reactive oxygen species (ROS), including superoxide anion (O_2_^−^), hydrogen peroxide (H_2_O_2_), hydroxyl radical (·OH), and singlet oxygen (^1^O_2_), are important signaling molecules in plant responses to stresses, but the overaccumulation of ROS leads to oxidative stress if unchecked [41]. In our transcriptome analysis, 53 DEGs were involved in ‘Antioxidant activity’ (Fig. 3B). To further investigate the expression of these antioxidant genes, the expression of representative genes was evaluated by RT-qPCR. Consistent with the results of transcriptome analysis, these antioxidant genes were differentially expressed in ‘Sopin’ and ‘Charging’ (Fig. 5A and Table S3). In particular, *superoxide dismutases* (*SOD1A*, *SOD1B*, and *SOD2B*) and *catalases* (*CATa*, *peroxisomal CAT*, and *CP*) displayed higher transcript levels in ‘Sopin’ than in ‘Charging’. However, the transcription levels of *PODc2*, *pmPOD2*, and *CCP* were much higher in ‘Sopin’ than in ‘Charging’ under dehydration conditions, while the levels of *POD2B* and *POD2C* were significantly lower (Fig. 5A).

Considering that SOD catalyzes the dismutation of O_2_^−^ to H_2_O_2_ and O_2_ and that CAT and POD catalyze the scavenging of H_2_O_2_ [42], the contents of O_2_^−^ and H_2_O_2_ in ‘Sopin’ and ‘Charging’ were also measured. Under normal conditions, the difference in O_2_^−^ content between ‘Sopin’ and ‘Charging’ was insignificant. After dehydration treatment, the concentration of O_2_^−^ in ‘Charging’ increased dramatically, which was 2.6-fold of that under normal conditions. However, the O_2_^−^ content in ‘Sopin’ increased only slightly by 0.4-fold (Fig. 5B). O_2_^−^ in leaves and roots was observed by staining with nitro-blue tetrazolium (NBT). The results showed that only a small part of the leaf tip was dyed blue, while no obvious signal was found in most parts of the leaves and roots under normal conditions. However, a strong blue color was observed under dehydration conditions. In both leaves and roots, the color of ‘Charging’ was darker than that of ‘Sopin’ (Fig. 5C), suggesting that the O_2_^−^ content was lower in ‘Sopin’ under stress conditions. The concentrations of H_2_O_2_ in ‘Sopin’ and ‘Charging’ were 2.1 and 2.4 µM/g, respectively, under normal conditions. However, those were 5.7 and 4.8 µM/g, respectively, under stress conditions (Fig. 5D). After staining H_2_O_2_ with 3,3’-diaminobenzidine (DAB), it could be observed that the color of H_2_O_2_ under stress was darker than that under normal conditions, and the color of ‘Charging’ was slightly lighter than that of ‘Sopin’ (Fig. 5E), suggesting that the H_2_O_2_ content was higher in ‘Sopin’ under stress conditions.

The difference in O_2_^−^ and H_2_O_2_ contents in ‘Sopin’ and in ‘Charging’ under dehydration conditions seemed in line with the expression of genes coding SOD, CAT, or POD. To further confirm that this difference was attributable to these antioxidant enzymes, their enzyme activities were evaluated. Compared with normal conditions, the activities of SOD, CAT, or POD increased after dehydration treatment. While the activity of SOD in ‘Charging’ increased by 0.5-fold, that in ‘Sopin’ increased by 2.1-fold. Although the activity of CAT in ‘Sopin’ was higher than that in ‘Charging’ under stress conditions, the activity of POD in ‘Sopin’ was lower (Fig. 5F). ROS have dual functions in plant cells: causing oxidative stress and acting as signaling molecules, especially the signaling roles of H_2_O_2_ in plant responses to environmental stresses, e.g., stomatal closure and root hair growth [42]. To investigate the oxidative damage caused by dehydration treatment, the content of malondialdehyde (MDA), an end product of lipid oxidation and an indicator of membrane damage caused by oxidative stress [39], was determined. The results showed that the concentration of MDA in ‘Sopin’ was lower, which was only 73% of that in ‘Charging’ under dehydration conditions (Fig. 5G), suggesting that the oxidative damage of the ‘Sopin’ membrane was weaker than that of ‘Charging’ under dehydration conditions. These results suggest that ‘Sopin’ could maintain a better balance between ROS-dependent signal transduction and protection against oxidative damage.

#### Transcriptional and posttranscriptional regulation in the stress response

**Fig. 6:**
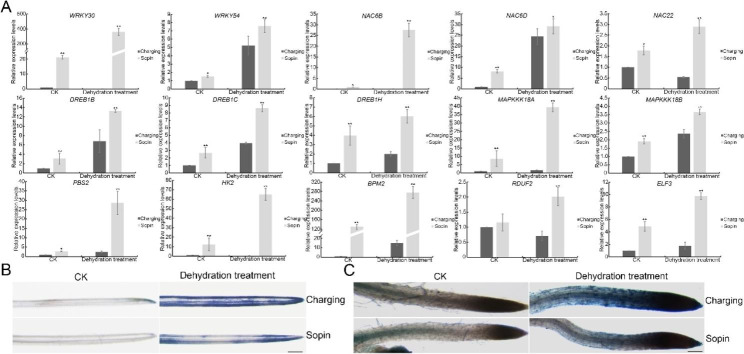
Expression of transcription factors, kinases, E3 ubiquitin ligases, and ABA-related genes. (**A**) Relative expression levels of representative transcription factors, kinases, E3 ubiquitin ligases, and ABA-related genes under dehydration treatment by RT-qPCR. *, *P* < 0.05 and **, *P* < 0.01 by Student’s *t*-test compared with gene expression levels in ‘Charging’. Data represent means ± SDs from three replicates. (**B**) The leaves stained by trypan blue. Bar = 500 μm. (**C**) The roots stained by trypan blue. Bar = 200 μm

Transcription factors, kinases, E3 ubiquitin ligases, and the phytohormone ABA play vital roles in drought stress signal transduction [4]. Among these DEGs potentially related to drought stress in our transcriptome analysis, 14 transcription factors, 12 kinases, and 8 E3 ubiquitin ligases were identified (Table S3). To further investigate the expression of these genes, the transcript levels of representative genes were checked by RT-qPCR. Consistent with the results of transcriptome sequencing, the expression levels of drought stress-related transcription factors of the WRKY family (WRKY30 and WRKY54), NAM ATAF1,2 and CUC2 (NAC) family (NAC6B, NAC6D, and NAC22), and dehydration-responsive element binding (DREB) family (DREB1B, DREB1C, and DREB1H) were significantly higher in ‘Sopin’ than in ‘Charging’, regardless of normal or dehydration conditions (Fig. 6A), suggesting that transcriptional regulation might be one of the adaptation mechanisms for ‘Sopin’. The expression of osmotic stress-activated protein kinase signaling cascades, MAP kinase kinase kinases (MAPKKK18A, and MAPKKK18B) and MAP kinase kinase (MAPKK) (PBS2), as well as cytokinin receptor kinase (HK2), increased substantially and were much higher in ‘Sopin’ (Fig. 6A). BTB-POZ AND MATH DOMAIN 2 (BPM2) and RING AND DOMAIN OF UNKNOWN FUNCTION 1117 2 (RDUF2), two E3 ubiquitin ligases that participate in ABA-mediated drought stress, were expressed at higher levels in ‘Sopin’ under dehydration treatment. In addition, *EARLY FLOWERING 3* (*ELF3*), which regulates ABA-modulated stomatal closure, also had higher expression in ‘Sopin’ (Fig. 6A), indicating that posttranscriptional regulation, including protein phosphorylation, proteasome-mediated protein degradation and the hormone ABA, also played roles in the ‘Sopin’ response to drought stress.

The higher expression of these transcription factors, kinases, and E3 ligases, as well as the regulation of osmolyte metabolism and ROS homeostasis, demonstrate that ‘Sopin’ adapts to drought stress, especially transient dehydration treatment, by multiple mechanisms. To further confirm that ‘Sopin’ has a higher adaptability to dehydration, the leaves and roots from dehydration-treated ‘Sopin’ and ‘Charging’ seedlings were stained with trypan blue, an indicator of cell death. Under normal conditions, leaves and roots from seedlings of both varieties were hardly stained. However, they were stained blue after dehydration treatment. Compared with those of ‘Charging’, the leaves and roots of ‘Sopin’ had less staining (Fig. 6B C). These data indicate that ‘Sopin’ is more tolerant to dehydration and suffers less cell damage.

## Discussion

The high degree of genetic diversity within the population of perennial ryegrass is an important resource [10], and the characterization of the genetic diversity of drought tolerance is the first step to utilize perennial ryegrass germplasm for drought resistance improvement. Because plant drought resistance is a complex characteristic closely related to the severity and duration of drought stress, plants can adapt to drought stress by different strategies, e.g., they can choose to survive by stopping growth or continue to grow slowly under stress conditions [43]. To fully understand the genetic diversity of the perennial ryegrass response to both extreme and moderate droughts, we evaluated the genetic diversity of drought resistance in 18 perennial ryegrass varieties. Our results demonstrated that ‘Sopin’ and ‘Banfield’ performed best under extreme or moderate drought conditions, respectively. The correlation between traits related to extreme stress and traits related to moderate stress was very low. Intriguingly, ‘Lightning’ was one of the varieties with the highest survival rate under extreme stress conditions but was also one of the varieties performing worst under moderate stress. ‘Pinnacle III’ had the highest relative growth rate under moderate stress of 50% SRWC but was not among the best varieties in terms of the survival rate under extreme stress. This finding is consistent with a previous report that increasing survival under severe drought does not indicate an improvement in performance under mild drought. Conversely, sustained growth under temporary mild drought conditions may threaten survival under long-term extreme drought stress [14]. The excellent performance under stress and the highest PCA ranking value of ‘Sopin’ suggest that it can be used in areas that often encounter drought stress, while ‘Charging’ with the lowest PCA ranking value is only suitable for areas without drought episodes.

To make better use of germplasm resources, it is essential to understand the genetic determinants and underlying mechanisms. Our transcriptome analysis demonstrated that 499 DEGs were involved in ‘Carbohydrate metabolism’, and 90 DEGs were particularly related to starch and sucrose metabolism. Starch is the major carbohydrate storage in plants and a key molecule in mediating the response to drought stress [27]. Under drought conditions, plants remobilize starch to provide energy and carbon when photosynthesis is limited. Additionally, the released maltose and other derived metabolites, such as glucose, fructose, and trehalose, are important osmolytes [44]. Osmotic stress-induced starch degradation is mediated by amylases, i.e., BAM and AMY. Mutants lacking these enzymes are sensitive to osmotic stress [27]. Other enzymes involved in soluble sugar metabolism are also important for plant drought resistance. For example, TPS is a key enzyme involved in the synthesis of trehalose [28], and its expression in many plant species improves drought resistance [45, 46]. HK phosphorylates glucose and confers plant drought resistance by modulating glucose homeostasis [30]. Our results showed that the expression of these enzymes was higher in ‘Sopin’, while CslA, which catalyzes the biosynthesis of insoluble cellulose and hemicellulose, was downregulated. Fructans are one of the main constituents of water-soluble carbohydrates in perennial ryegrass and are involved in drought resistance [35, 47, 48]. The accumulation of fructans was determined by FTs, FEHs,and the availability of substrate molecule sucrose [36, 49]. The expression of *1-SSTa* was slightly higher while that of *1-SSTb* and *1-SSTc* were lower in ‘Sopin’ under dehydration conditions, whereas the expression of *FEH* was lower in ‘Sopin’ relative to ‘Charging’ under dehydration conditions. Possibly, this was because fructosyltransferases (FTs) are transcriptional and posttranslational regulated and the fructan concentration is not only determined by the expression levels of FTs [32, 50]. Consistent with the expression of these enzymes, the soluble sugar content in ‘Sopin’ was 1.5-fold that in ‘Charging’ under stress conditions. Proline is another osmolyte for osmotic adjustment under stress conditions [39, 51]. However, the proline content in ‘Sopin’ was lower than that in ‘Charging’, probably due to the lower expression of P5CS under dehydration conditions. These findings imply that the response strategies of ‘Sopin’ and ‘Charging’ to drought stress are different in osmolyte metabolism.

As signaling molecules or cause oxidative stress, ROS play essential roles in plant adaptation to abiotic stress [41]. Drought and other stresses cause the accumulation of O_2_^−^, which is subsequently converted to H_2_O_2_. H_2_O_2_ is an important signaling molecule that mediates various types of acclimation signal transduction, such as stomatal closure, gene expression, root hair growth, and systemic stress signaling of the whole plant [20, 42, 52], whereas the overproduction of ROS causes oxidative damage [7]. Mutants impaired in ROS production or oxidative damage scavenging were found to be more sensitive to abiotic stresses and vice versa [41]. SOD represents the first line of antioxidant defense by rapidly converting O_2_^−^ and decreasing the formation of highly toxic ·OH. Furthermore, SOD is required to rapidly induce the H_2_O_2_ signature and trigger adaptive responses under stress conditions [53]. Overexpression of SOD improves drought resistance [54]. Once the signaling is completed, other enzymatic antioxidants (e.g., CAT and POD) scavenge H_2_O_2_ to basal level [42]. In ‘Sopin’, higher gene expression and enzyme activity of SOD and lower O_2_^−^ content were found under dehydration conditions. Different from the higher transcription levels of *PODc2*, *pmPOD2*, and *CCP*, the transcription levels of *POD2B* and *POD2C* were lower in ‘Sopin’ than in ‘Charging’. Consequently, POD activity was lower and H_2_O_2_ content was higher in ‘Sopin’ under dehydration treatment. Consistent with the role of H_2_O_2_ in modulating stomatal closure, the water loss rate by transpiration in ‘Sopin’ was the lowest. The low level of MDA suggested that the membrane of ‘Sopin’ suffered less oxidative damage. Soluble sugars also mitigate oxidative damage by scavenging ·OH and other ROS [47]. The differential expression and activity of antioxidant enzymes and the accumulation of more soluble sugars and less MDA in ‘Sopin’ indicate that ‘Sopin’ maintains a better balance between maintaining ROS-modulated signal transduction and alleviating oxidative damage under stress conditions.

**Fig. 7:**
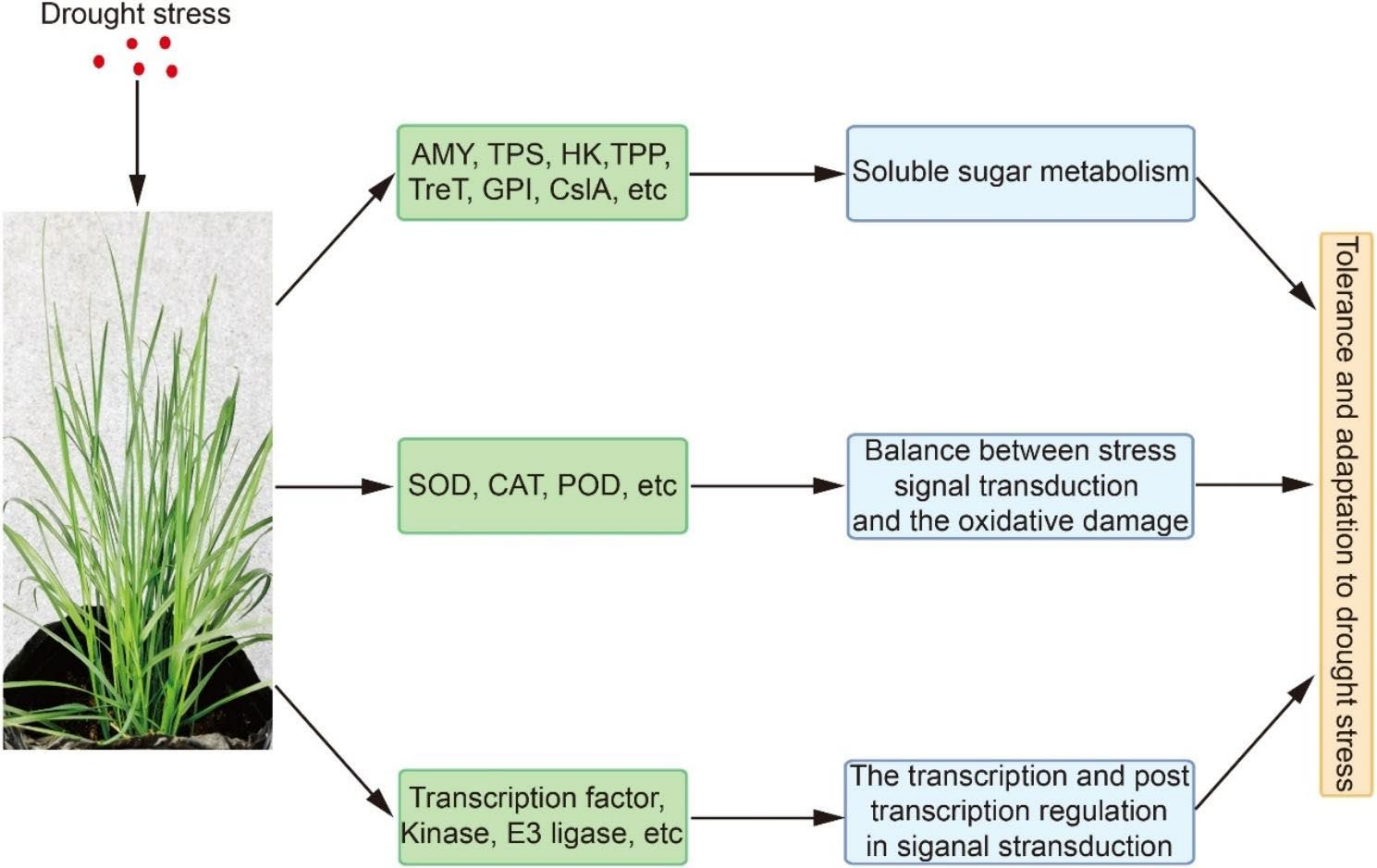
Model for the mechanisms by which ‘Sopin’ adapts to drought stress

Plants adapt to drought stress by changing gene and metabolism patterns after perceiving stress signals. Transcription factors, kinases, E3 ubiquitin ligases, and ABA play important roles in regulating gene expression, protein activity, and physiological metabolism [4]. WRKY, NAC, and DREB transcription factor families are important regulators of gene expression in response to drought stress. For example, several WRKYs mediate the ABA response and drought tolerance by regulating the expression of *ABI5* or *RD29A* [55, 56]; DREB transcription factors enhance drought tolerance by activating the expression of downstream genes (e.g., *RD29A* and *RD29B*) [57, 58]; and NACs specifically bind to a drought-responsive *cis*-element to activate the expression of many drought-responsive genes [59]. Overexpression of these transcription factors, such as NAC6 and DREB1B, improves plant drought tolerance [57, 60]. The high expression of these transcription factor family members in ‘Sopin’ indicates that transcriptional regulation is one of the adaptation mechanisms. Phosphorylation and ubiquitination are important posttranscriptional regulatory mechanisms. MAP kinase cascades are observed in the signal transduction of ABA-modulated stomatal closure and drought resistance [20, 61]. Histidine kinases have been shown to positively regulate drought and ABA responses [62]. E3 ubiquitin ligase is an important component of the proteasome degradation system. BPMs, substrate-binding adaptors of Cullin3-based E3 ligase, affect stomatal closure by modulating the degradation of the ABA negative regulator ATHB6 [63]. Suppression of RING-DUF1117 E3 ubiquitin ligases RDUF1 and RDUF2 reduces ABA-mediated drought resistance [64]. ELF3 regulates stomatal closure by specifically affecting the ABA response [65]. The high expression of MAPKKK18s, PBS2, HK2, BPM2, RDUF2, and ELF3 in ‘Sopin’ demonstrated that posttranscriptional regulatory mechanisms and the phytohormone ABA were also responsible for ‘Sopin’ tolerance to drought stress. Therefore, ‘Sopin’ adapts to drought stress by multiple mechanisms. Specifically, AMY, TPS, HK, and other starch and sugar metabolic enzymes were highly expressed, while CslA was expressed at low levels under drought conditions, promoting starch degradation and soluble sugar accumulation. The expression and activity of antioxidant enzymes (SOD, CAT, and POD) were differentially regulated to minimize oxidative damage while maintaining ROS signal transduction. Furthermore, drought stress-related transcription factors, kinases, and E3 ubiquitin ligases were upregulated to facilitate ABA and stress signal transduction (Fig. 7).

## Conclusion

We found a large variation in drought stress resilience in the 18 perennial ryegrass varieties studied. The survival capacity of perennial ryegrass species under severe drought stress differed from that under moderate drought stress. ‘Sopin’, with superior performance under both extreme and moderate stress conditions, was one of the best-performing varieties. ‘Sopin’ adaptation to drought stress appears to be achieved through the activation of sophisticated mechanisms, possibly including transcriptional and posttranscriptional regulation, the hormone ABA, as well as the adjustment of osmolyte metabolism and ROS homeostasis. Although a complete understanding of the detailed functions of the identified stress-regulated genes has yet to be achieved, our research provides useful information for understanding the genetic diversity of the perennial ryegrass response to drought stress and the underlying adaptation mechanisms.

## Materials and methods

### Plant materials and growth conditions

Twenty-one varieties of perennial ryegrass commonly used for lawn construction in China were collected from the Rytway Seed Company and Barenbrug Company. Among them, 18 varieties that did not show delayed seed germination and uneven growth with other varieties were used in this study. Perennial ryegrass seeds were sown onto quartz and placed in a growth room at 25 °C with a 12-h light/12-h dark cycle, 65% relative humidity, and 200 µmol m^–2^ s^–1^ photosynthetically active radiation for germination and growth. One-week-old seedlings of similar size were transferred to matrix soil (Pindstrup Sphagnum moss peat and vermiculite, 1:1 of v/v) for further growth in the growth room.

### Drought resistance analysis of perennial ryegrass varieties

The soil water content was determined by a soil moisture meter (Saiyasi Technology Co., Ltd, SYS-SD). After germination, one-week-old seedlings of similar size were transferred to plant pots containing 50 g matrix soil. Each pot contained 16 seedlings, and plants were irrigated daily. After growing for another 10 days, the seedlings of each variety were used for stress experiments. For extreme drought treatment, twelve pots of plants for each variety had watering withheld until the soil relative water content (SRWC) decreased to 30%. Then, water was added daily to maintain this soil moisture for 2 weeks. After treatment, seedlings were irrigated to allow recovery for 10 days. Alternatively, 12 pots of plants for each variety had watering withheld until SRWC decreased to 20% and maintained this soil moisture for 10 days, and then seedlings were recovered for 10 days. Photographs and relative water content (RWC) were taken before and after drought treatment and 10 days after rewatering. Four independent pots of plants for each variety were used for the measurement of RWC with the formula $\text{R}\text{W}\text{C}=\frac{\text{F}\text{W}-\text{D}\text{W}}{\text{T}\text{W}-\text{D}\text{W}} \times \text{100\%}$. FW, TW, and DW represent the fresh weight, turgid weight, and dry weight of seedlings, respectively. Three independent repeats of these experiments were performed. For the moderate drought treatment, 21 pots of plants for each variety were randomly assigned to three groups. Seedlings of the first group (control group) were irrigated daily to maintain normal soil moisture (75% SRWC). Seedlings of the second and third groups had water withheld until SRWC decreased to 50% or 40%, respectively, and then irrigated daily to maintain the soil moisture. When the SRWC of the third group decreased to 40% for 2 weeks, more than 100 seedlings for each variety under normal or stress conditions were used to measure the relative growth rate, and four independent pots of plants for each variety were used to score the relative aboveground weight. Three independent repeats of this experiment were performed.

For dehydration treatment, 7-day-old seedlings were dehydrated on wet filter paper in petri dishes until 50% fresh weight was lost and then incubated for 2 h in sealed plastic bags as previously described [26]. Samples (whole plants, leaves, or roots) from seedlings treated with or without dehydration were collected for further analyses.

Detached leaves from 17-day-old seedlings were exposed to air in a growth room and weighed at the indicated time points, and then the water loss rate was scored according to a previous description [26]. The whole experiment was carried out three independent times, and each experiment was performed with three biological replicates (each biological replicate consisted of approximately 20 leaves) for each variety.

### PCA

Principal component analysis (PCA) was performed using SPSS software as previously described with traits of the water loss rate (WLR) of detached leaves, the ratio of relative water content after rewatering to that before stress treatment (RWC), survival rates under 30% and 20% SRWC conditions (SR30 and SR20), relative growth rates under 50% and 40% SRWC conditions (GR50 and GR40), and relative aboveground weights under 50% and 40% SRWC (W50 and W40) [6]. The PCA value was scored by the formula PCA =${\sum }_{j=1}^{n}\left[PCj*contribution of PCj\right(\%\left)\right]$*j*=1; 2; 3;…; n. Principal components with initial eigenvalues > 1 were selected for PCA rank calculation.

### Transcriptome analysis

Total RNA from dehydration-treated ‘Sopin’ and ‘Charging’ seedlings was extracted using the RNeasy Mini Kit (Invitrogen), and DNA was cleaned by DNase I (New England Biolabs). RNA sequencing and analysis were carried out by a commercial gene sequencing company, Annoroad Gene Technology Company. Briefly, after evaluation of the RNA purity and integrity by using a NanoPhotometer® Spectrophotometer (IMPLEN) and Bioanalyzer 2100 system (Agilent Technologies), RNA-seq libraries were constructed with a total amount of 1 µg RNA per sample using the NEB Next® UltraTM RNA Library Prep Kit and sequenced on the Illumina HiSeq platform to generate high-quality paired-end reads of 200–300 bp in length. More than 6.7 Gb of raw data for each sample were collected. After removing reads containing poly-N or adapters and low-quality reads from raw data, the obtained clean reads were assembled using Trinity (v. 2.3.3.10) as previously described [66]. Gene expression levels were calculated as reads per kilobase of transcript per million mapped reads (RPKM). DEGs were determined using the DESeq R package (1.10.1) with criteria of fold change ≥ 2, p value ≤ 0.005, and adjusted p value ≤ 0.01. Gene function was annotated based on the databases of NR (NCBI nonredundant protein sequences), Pfam (Protein family), KOG/COG/eggNOG (Clusters of Orthologous Groups of proteins), Swiss-Prot (a manually annotated and reviewed protein sequence database), KEGG (Kyoto Encyclopedia of Genes and Genomes developed by Kanehisa Laboratories [67]) and GO (Gene Ontology). Enrichment analyses were performed using the Enrichment Analysis module (Fisher’s Exact Test) in BLAST2GO with a Q-value ≤ 0.05. Three biological replicates of each variety were used for the transcriptome analysis (approximately 1.0 g 7-day-old seedlings were used to extract RNA for each biological replicate). The raw data were uploaded to the SRA database of NCBI under accession number PRJNA902027.

### RT-qPCR

Total RNA was extracted, and DNA was cleaned. cDNAs were synthesized by using SuperScript III reverse transcriptase (Takara). RT-qPCR was performed on a Real-Time PCR System (Bio-Rad) by using *TEF1* and *eEF1A* as internal controls. The primers used in this study are presented in Table S4. Three biological replicates for each sample were performed, and approximately 1.0 g ‘Sopin’ or ‘Charging’ seedlings were used for each biological replicate.

### Soluble sugar and proline content determination

For soluble sugar content, 1.0 g of seedlings was powdered and homogenized in 10 ml of 80% ethanol. After incubation at 80 °C for 30 min, the samples were centrifuged at 10,000 × *g* for 15 min. The centrifugation was repeated, and the volume of the obtained supernatant was fixed at 15 ml. A total of 5 ml of anthrone-H_2_SO_4_ was added to 2 ml of supernatant that was diluted 10 times with distilled water. After boiling for 10 min, the absorbance at 620 nm was measured. The soluble sugar content was calculated based on a standard curve. Four biological replicates for each sample were performed, and 1.0 g ‘Sopin’ or ‘Charging’ seedlings were used for each biological replicate.

The proline content was assayed as previously described [68]. One gram of seedlings was powdered and homogenized in 10 ml of 3% C_7_H_6_O_6_S·2H_2_O and then boiled for 15 min. One milliliter of distilled water, 2 ml of glacial acetic acid, and 2 ml of ninhydrin reagent were added sequentially into 1 ml of extract. Samples were boiled for 30 min and then mixed with an equal volume of toluene. The absorbance of the organic layer at 546 nm was measured. The proline content was calculated based on a standard curve. Four biological replicates for each sample were performed, and 1.0 g ‘Sopin’ or ‘Charging’ seedlings were used for each biological replicate.

### O_2_^−^, H_2_O_2_ and MDA contents

The O_2_^−^ content was analyzed as previously described [69]. One gram of seedlings was powdered and homogenized in 10 ml of 65 mM PBS buffer (pH 7.8). Two milliliters of supernatant was mixed with 1.5 ml of PBS buffer (pH 7.8) and 0.5 ml of 10 mM NH_2_OH·HCl. The mixture was incubated at 25 °C for 20 min. Then, equal volumes of 17 mM C_6_H_7_NO_3_S and 7 mM C_10_H_9_N were added and incubated at 30 °C for 30 min. The absorbance at 530 nm was measured, and the O_2_^−^ content was calculated based on the standard curve. Four biological replicates for each sample were performed, and 1.0 g ‘Sopin’ or ‘Charging’ seedlings were used for each biological replicate. For staining O_2_^−^ with nitro-blue tetrazolium (NBT), leaves or roots were vacuumed with 1 mg/ml NBT solution for 15 min. Samples were boiled for 2 min, destained with 75% ethanol and photographed using a microscope. Two independent replicates of this staining experiment were performed.

The H_2_O_2_ content was determined according to a previous description [47]. One gram of seedlings was powdered and homogenized in 10 ml of 5% trichloroacetic acid. After 4°C 12000 × *g* centrifugation for 20 min, equal volumes of 100 mM potassium phosphate buffer (pH 7.0) and 1 M KI were added and incubated at 4°C in the dark for 10 min. The absorbance at 390 nm was measured, and the concentration of H_2_O_2_ was scored based on a standard curve. Four biological replicates for each sample were performed, and 1.0 g ‘Sopin’ or ‘Charging’ seedlings were used for each biological replicate. For staining H_2_O_2_ with 3,3’-diaminobenzidine (DAB), leaves or roots were vacuumed with 1 mg/ml DAB solution for 10 min. Samples were incubated in the dark for 8 h, destained with 75% ethanol and photographed using a microscope. Two independent replicates of this staining experiment were performed.

The malondialdehyde (MDA) content was determined according to a previous description [7]. One gram of seedlings was powdered and homogenized in 10 ml of 50 mM PBS buffer (pH 7.8). Then, 10 ml of 0.5% thiobarbituric acid was added and boiled for 10 min. The absorbance at 450, 532, and 600 nm was measured, and the content of MDA was scored by the formula MDA (M/g) $=\frac{{6.452 \times (A532 - {\rm{A}}600) - 0.559 \times A450}}{{{{\rm{V}}_{\rm{s}}} \times {{\rm{F}}_{\rm{w}}}}} \times {{\rm{V}}_{\rm{t}}}$, where V_t_ is the total volume of the reaction mixture, V_s_ is the volume for measurement, and F_W_ is the sample weight. Three biological replicates for each sample were performed, and 1.0 g ‘Sopin’ or ‘Charging’ seedlings were used for each biological replicate.

### Antioxidant enzyme activity

One gram of seedlings was powdered and homogenized in 10 ml of 50 mM PBS buffer (pH 7.8). After 4 °C 12,000 × *g* centrifugation for 15 min, the supernatant was used. For superoxide dismutase (SOD) activity, 0.3 ml of supernatant was mixed with 3 ml of solution containing 25 mM PBS (pH 7.8), 10 µM EDTA, 13 mM methionine, 75 µM NBT and 2 µM riboflavin. The mixture was illuminated for 15 min, and the absorbance at 560 nm was measured. The reaction without supernatant was used as a control. The activity of SOD was scored by the formula SOD (U·g^− 1^·h^− 1^) $=\frac{(\text{A}_{\text{0}}-\text{A}_{\text{s}}) \text{V}_{\text{t}} \times \text{60}}{\text{A}_{\text{0}} \times 0.5 \times \text{F}_{\text{w}} \times \text{V}_{\text{s}} \times \text{t}}$, where A_0_ is the absorbance of control samples, A_s_ is the absorbance of samples, V_t_ is the total volume of the reaction mixture, V_s_ is the volume for measurement, F_W_ is the sample weight, and t is the illumination duration. For catalase (CAT) activity, 0.5 ml of supernatant was mixed with 1 ml of 1 mM Tris-HCl (pH 7.0), 1.5 ml of distilled water, and 0.2 ml of 200 mM H_2_O_2_. The absorbance at 240 nm was immediately measured for 2 min. The boiled supernatant was used as a control. The activity of CAT was scored by the formula CAT (U·g^− 1^·min^− 1^) $=\frac{(\text{A}_{\text{0}}-\text{A}_{\text{s}}) \times \text{V}_{\text{t}}}{0.1 \times \text{V}_{\text{s}}\times \text{t} \times {\text F}_\text{w}}$, where A_0_, A_s_, V_t_, V_s_, and F_W_ are the same as those described in the SOD activity analysis and t is the duration of the measurement. For peroxidase (POD) activity, 1 ml of supernatant was mixed with 1 ml of 0.1% guaiacol, 6.9 ml of distilled water, and 1 ml of 30% H_2_O_2_. After 10 min of incubation, 0.2 ml of 5% metaphosphoric acid was added to terminate the reaction, and the absorbance at 470 nm was measured. The reaction without supernatant was used as a control. The activity of POD was scored by the formula POD(µg·g^− 1^·min^− 1^)$\text{=}\frac{ (\text{X}-\text{X}_{\text{0}}) \times \text{V}_{\text{t}}}{\text{F}_{\text{w}} \times \text{V}_{\text{s}}\times \text{t}}$, where X is the content of tetra-o-methoxyphenol in the reaction mixture, X_0_ is the content of guaiacol in the control sample, V_t_, V_s_ and F_W_ are the same as those described in the SOD activity analysis, and t is the reaction duration. These experiments were performed with three biological replicates for each sample, and 1.0 g ‘Sopin’ or ‘Charging’ seedlings were used for each biological replicate.

### Trypan blue staining

Leaves or roots were vacuumed with 0.1% trypan blue for 15 min and stained for another hour. Then, samples were boiled for 2 min, destained with 75% ethanol and photographed using a microscope. Two independent replicates of this staining experiment were performed.

### Statistical analysis

The data presented in plots of this study represent means ± SDs (standard deviation). The asterisk above the column in the figures represents a significant difference compared to the control at the *P* < 0.05 or *P* < 0.01 level by Student’s *t*-test.

### Electronic supplementary material

Below is the link to the electronic supplementary material.

Supplementary Material 1

Supplementary Material 2

Supplementary Material 3

Supplementary Material 4

Supplementary Material 5

## Data Availability

Transcriptome sequencing data are available in the SRA database of the National Center for Biotechnology Information (NCBI) under accession numbers BioSample SAMN31746585 - SAMN31746590 or BioProject PRJNA902027 (https://www.ncbi.nlm.nih.gov/bioproject/PRJNA902027).
