# Supplementary material for: The genetic variation in drought resistance in eighteen perennial ryegrass varieties and the underlying adaptation mechanisms
Source: BMC Plant Biol. 2023 Sep 26;23:451. doi: 10.1186/s12870-023-04460-z (PMC10521523; doi:10.1186/s12870-023-04460-z)
Supplement: Supplementary file 1 — Supplementary Material 1 [file 12870_2023_4460_MOESM1_ESM.pdf]

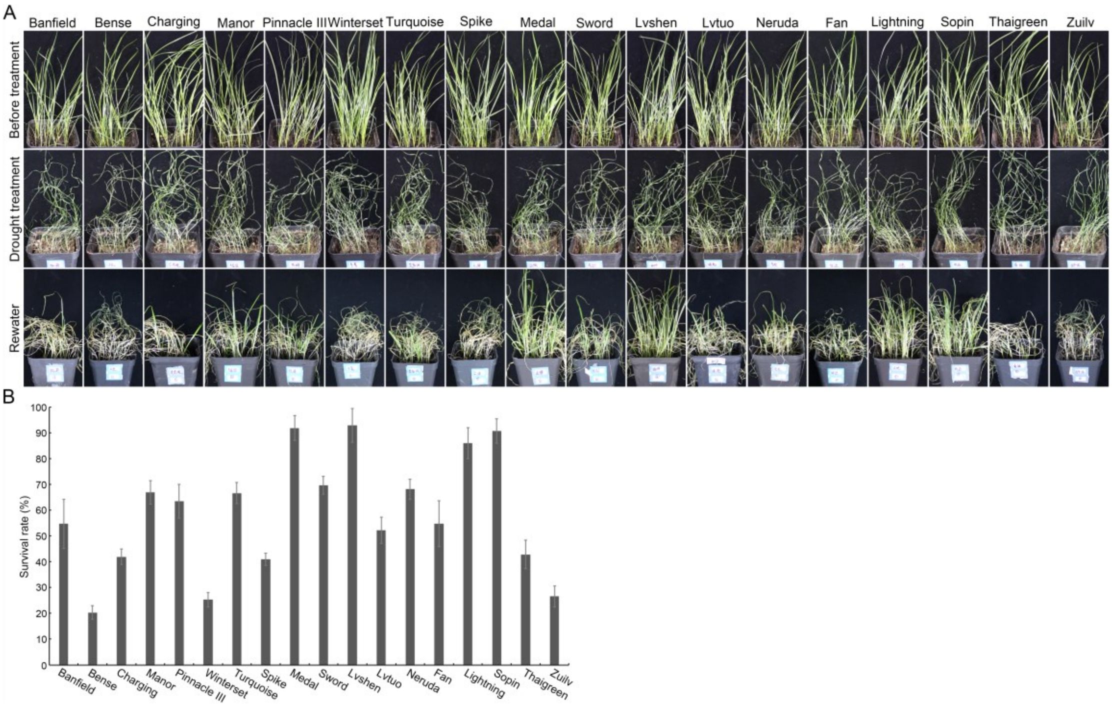

Fig. S1 The survival of perennial ryegrass varieties under 20% SRWC conditions. (**A**) Morphology of seedlings before and after the 20% SRWC treatment. Photographs were taken before and after 20% SRWC treatment and 10 days after rewatering. (**B**) Seedling survival rate. At least 120 seedlings for each variety were scored. Data represent means ± SDs.

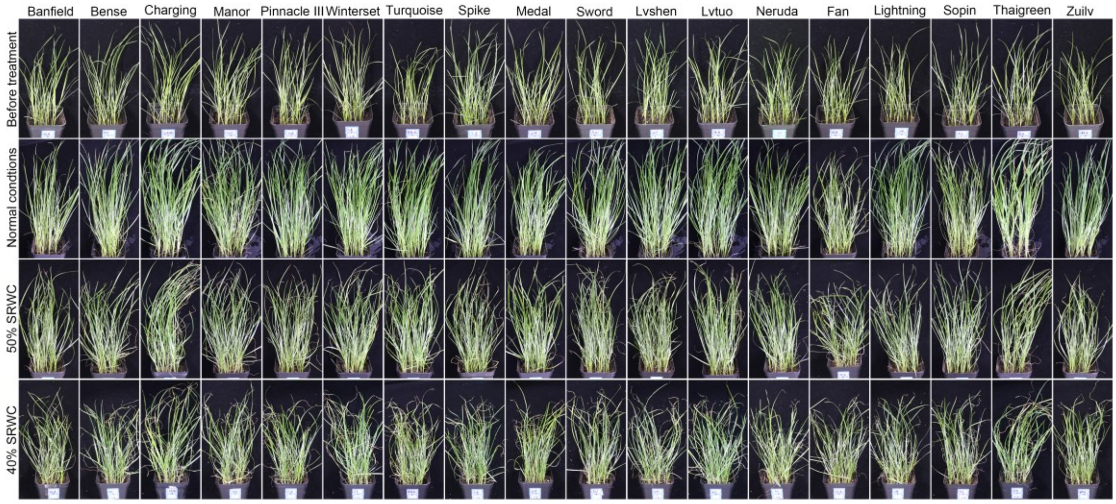

Fig. S2 Morphology of seedlings growing under normal or moderate drought conditions. Photographs were taken before and after 50% SRWC or 40% SRWC treatment.

A

KMO measure of sampling adequacy		0.603
Bartlett's test of sphericity	Approx. shi-square	94.827
	df	28
	Sig.	0.000

B

Principal component	Initial eigenvalues	
	Eigenvalue	Contribution ratio (%)
PC1	3.624	45.30
PC2	1.811	22.64
PC3	1.316	16.45
PC4	0.488	6.10
PC5	0.469	5.86
PC6	0.195	2.44
PC7	0.056	0.70
PC8	0.041	0.52

C

Traits	Component matrix$^{\alpha}$		
	PC1	PC2	PC3
WLR	-0.926	-0.250	0.119
RWC	0.304	0.689	0.436
SR30	0.490	0.606	0.350
SR20	0.451	0.614	-0.429
GR50	0.794	-0.321	-0.345
GR40	0.695	-0.121	-0.549
W50	0.737	-0.482	0.424
W40	0.757	-0.424	0.453

Fig. S3 Principle component analysis of 8 traits across these 18 perennial ryegrass varieties. (**A**) KMO and Bartlett's test on trait values. (**B**) The eigenvalue and contribution ratio of each principal component. (**C**) The component matrix$^{\alpha}$ of the first three principle components corresponding to each trait.

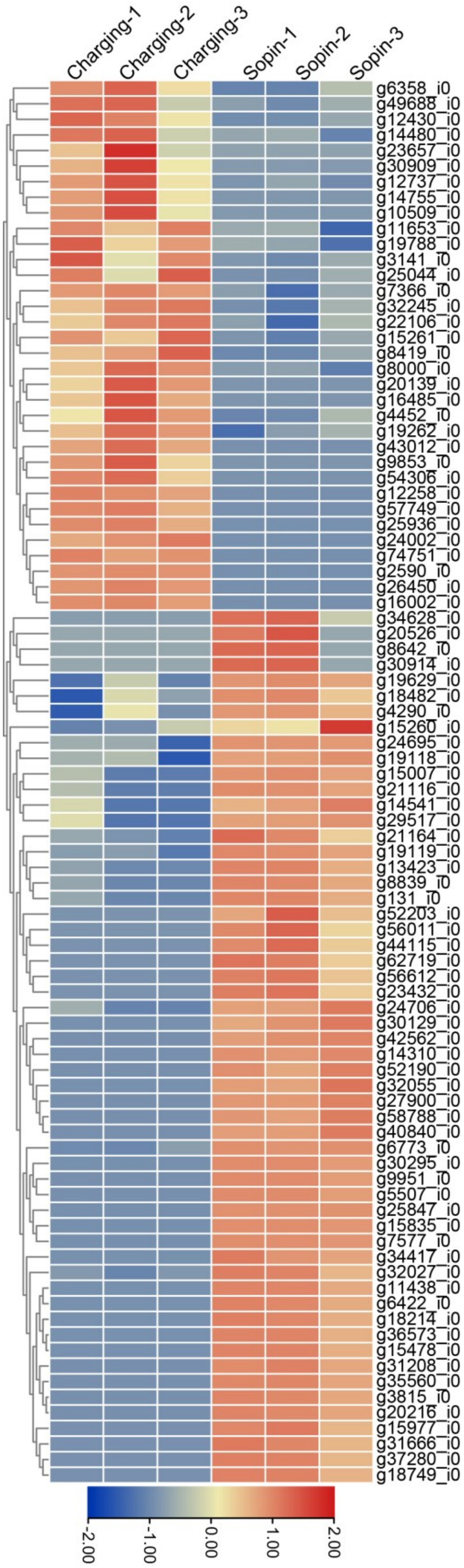

Fig. S4 Heatmap diagrams showing the expression pattern of DEGs involved in starch and sucrose metabolisms

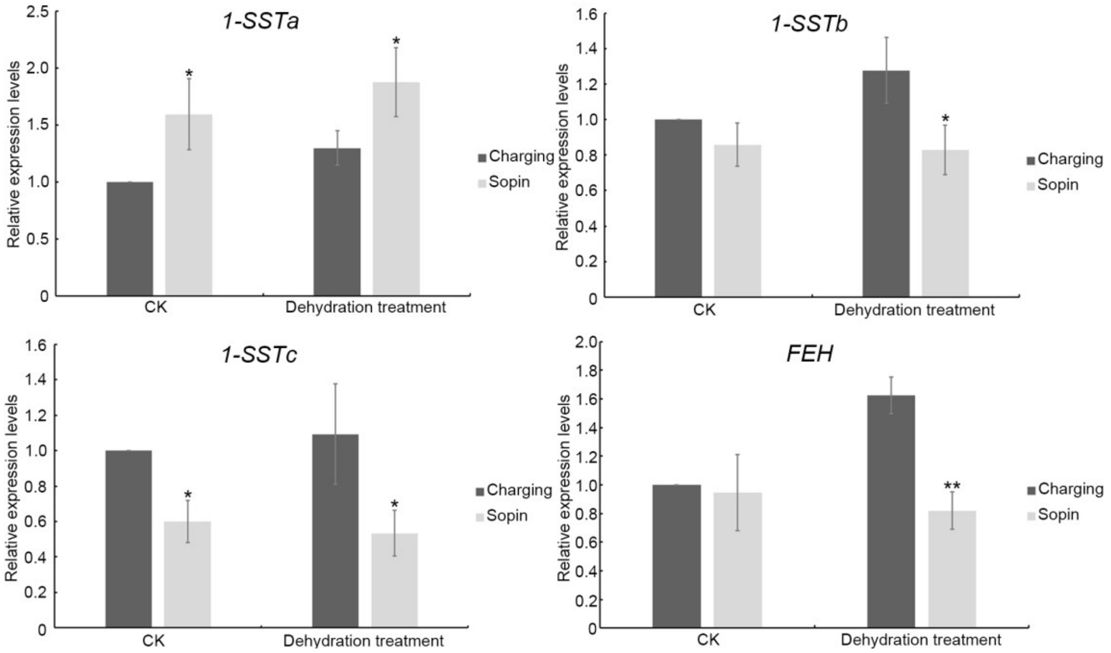

Fig. S5 Relative expression levels of SSTs and FEH that regulation frutcan accumulation. *, $P < 0.05$ and **, $P < 0.01$ by Student's t-test compared with gene expression levels in 'Charging'. Data represent means ± SDs from three replicates.

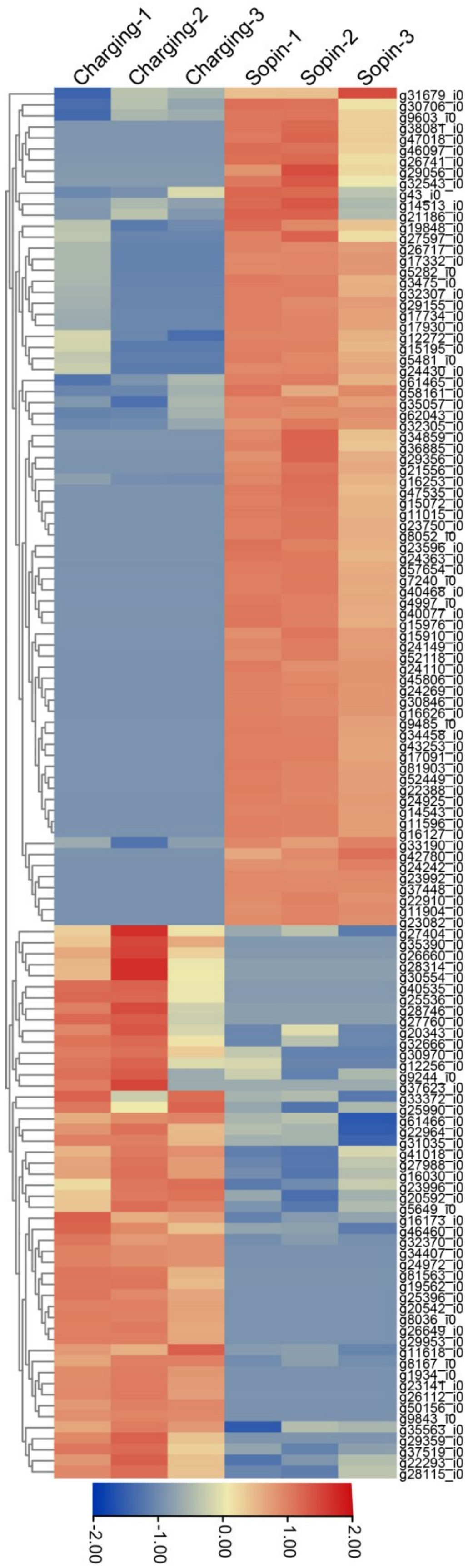

Fig. S6 Heatmap diagrams showing the expression pattern of DEGs involved in biosynthesis of amino acids

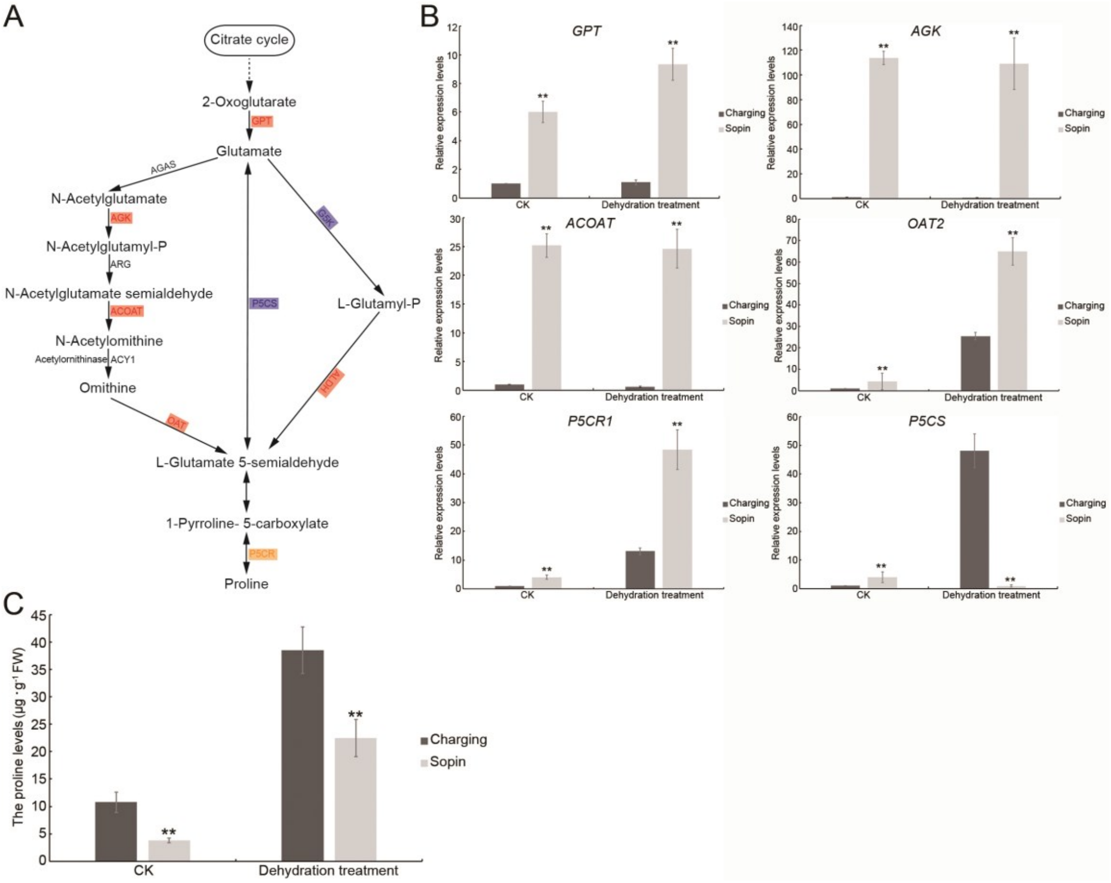

Fig. S7 The proline metabolism in ‘Sopin’ and ‘Charging’ under dehydration treatment. (**A**) The proline metabolic pathway based on the KEGG database (ko00330). In transcriptome analysis, enzymes upregulated in ‘Sopin’ are labeled with red boxes, enzymes downregulated are labeled with blue boxes, and the yellow box labeled enzymes are coded by genes that were both upregulated and downregulated in ‘Sopin’. (**B**) Relative expression levels of selected genes involved in proline metabolism by RT-qPCR. **, $P < 0.01$ by Student’s *t*-test compared with gene expression levels in ‘Charging’. Data represent means ± SDs from three replicates. (**C**) The concentration of proline. **, $P < 0.01$ by Student’s *t*-test compared with the content in ‘Charging’. Data represent means ± SDs from four replicates.
