# Supplementary material for: The genetic variation in drought resistance in eighteen perennial ryegrass varieties and the underlying adaptation mechanisms
Source: BMC Plant Biol. 2023 Sep 26;23:451. doi: 10.1186/s12870-023-04460-z (PMC10521523; doi:10.1186/s12870-023-04460-z)
Supplement: Supplementary file 4 — Supplementary Material 4 [file 12870_2023_4460_MOESM4_ESM.docx]

| **Table S3. DEGs that are involved in the plant response to drought stress** | | |
| --- | --- | --- |
| **Genes up-regulated in Sopin** | | |
| **Gene ID** | **Gene name** | **Description** |
| g48499_i0 | WRKY54 | Transcription factor; Regulation of response to water deprivation |
| g43700_i0 | WRKY30 | Transcription factor; Regulation of response to hydrogen peroxide |
| g36893_i0 | NAC6A | Transcription factor; Positive regulation of response to water deprivation |
| g32480_i0 | NAC6B | Transcription factor; Positive regulation of response to water deprivation |
| g32481_i0 | NAC6C | Transcription factor; Positive regulation of response to water deprivation |
| g50496_i0 | NAC6D | Transcription factor; Positive regulation of response to water deprivation |
| g43301_i0 | NAC22 | Transcription factor; Positive regulation of response to water deprivation |
| g11064_i0 | ERF59 | Transcription factor; Regulation of response to osmotic stress |
| g38459_i0 | DREB1H | Dehydration-responsive element-binding protein; Transcription factor |
| g27234_i0 | DREB1C | Dehydration-responsive element-binding protein; Transcription factor |
| g41924_i0 | DREB1B | Dehydration-responsive element-binding protein; Transcription factor |
| g5264_i0 | ATF1 | Transcription factor; Regulation of response to osmotic stress |
| g56078_i0 | MIC19 | Mitochondrial inner membrane; Regulation of response to osmotic stress |
| g12800_i0 | EXO70B2 | Exocyst complex component; Regulation of response to water deprivation |
| g63269_i0 | RBG7 | Regulation of alternative mRNA splicing, regulates response to water deprivation |
| g23432_i0 | TreT | Trehalose synthase; Glucose metabolic process |
| g6422_i0 | TREH3 | Glycogensynthase; Glycogen biosynthetic process |
| g34417_i0 | TPS1 | α, α-trehalose-phosphate synthase; Trehalose metabolism in response to stress |
| g3815_i0 | TPS2 | α, α-trehalose-phosphate synthase; Trehalose metabolism in response to stress |
| g62719_i0 | TPS3 | α, α-trehalose-phosphate synthase; Trehalose metabolism in response to stress |
| g52203_i0 | TPPe | Trehalose-phosphatase; Trehalose metabolism in response to stress |
| g24695_i0 | TPP7A | Trehalose-phosphatase; Trehalose biosynthetic process |
| g19119_i0 | TPP7B | Trehalose-phosphatase; Trehalose biosynthetic process |
| g19118_i0 | TPP7C | Trehalose-phosphatase; Trehalose biosynthetic process |
| g44115_i0 | MGAM | β-fructofuranosidase; Sucrose catabolic process |
| g11633_i0 | MAL11A | Maltose permease; Maltose metabolic process |
| g21449_i0 | MAL11B | Maltose permease; Maltose metabolic process |
| g37734_i0 | MAL11C | Maltose permease; Maltose metabolic process |
| g56011_i0 | HK I | Hexokinase; Glucose homeostasis |
| g20216_i0 | HK | Hexokinase; Glucose homeostasis |
| g56612_i0 | GYS1 | α-amylase; Carbohydrate catabolic process |
| g18482_i0 | GYS2 | α-amylase; Carbohydrate catabolic process |
| g8839_i0 | GBE2 | 1,4-α-α-branching enzyme; Glycogen biosynthetic process |
| g18749_i0 | EXG1 | Glucan exo-1,3-β-glucosidase; Carbohydrate metabolic process |
| g20666_i0 | EXG2 | Glucan exo-1,3-β-glucosidase; Carbohydrate metabolic process |
| g19629_i0 | AMY1 | 1,4-α-D-glucan glucanohydrolase; Carbohydrate metabolic process |
| g42562_i0 | AMY2 | 1,4-α-D-glucan glucanohydrolase; Carbohydrate metabolic process |
| g21164_i0 | AMY3 | 1,4-α-D-glucan glucanohydrolase; Carbohydrate metabolic process |
| g21116_i0 | AMY4 | 1,4-α-D-glucan glucanohydrolase; Carbohydrate metabolic process |
| g19661_i0 | AMY5 | 1,4-α-D-glucan glucanohydrolase;carbohydrate metabolic process |
| g18214_i0 | AMY6 | 1,4-α-D-glucan glucanohydrolase;carbohydrate metabolic process |
| g11438_i0 | AMY7 | 1,4-α-D-glucan glucanohydrolase;carbohydrate metabolic process |
| g14513_i0 | PFK3 | ATP-dependent 6-phosphofructokinase; Fructose 6-phosphate metabolic process |
| g7240_i0 | PFK | ATP-dependent 6-phosphofructokinase; Fructose 6-phosphate metabolic process |
| g15007_i0 | GPI | Glucose-6-phosphate isomerase; Carbohydrate catabolic process |
| g6773_i0 | 1-SSTa | Sucrose:sucrose 1-fructosyltransferase; Carbohydrate metabolic process |
| g24110_i0 | P5CR1 | Pyrroline-5-carboxylate reductase; L-proline biosynthetic process |
| g36460_i0 | OAT1 | Ornithine-oxo-acid transaminase; Arginine catabolic process to proline via ornithine |
| g24814_i0 | OAT2 | Ornithine-oxo-acid transaminase; Arginine catabolic process to proline via ornithine |
| g17091_i0 | GPT | Glutamate pyruvate transaminase; Alanine and proline catabolic process |
| g16578_i0 | ALDH | Aldehyde dehydrogenase; Penicillin biosynthetic process |
| g4997_i0 | AGK | N-acetyl-gamma-glutamyl-phosphate reductase; Arginine and proline biosynthetic process |
| g23596_i0 | ACOAT | Acetylornithine aminotransferase; Arginine and proline biosynthetic process |
| g14202_i0 | RZF1 | Regulation of proline biosynthetic process; Response to osmotic stress |
| g13541_i0 | PP2C1 | Protein phosphatase 2C. Cellular response to osmotic stress |
| g9861_i0 | SRK1 | Protein kinase; Cellular response to osmotic stress |
| g16159_i0 | SPM1 | Cellular response to osmotic stress; MAPK cascade |
| g6992_i0 | PBS2 | Activation of MAPK activity involved in osmosensory signaling pathway |
| g26761_i0 | MAPKKK18A | Osmotic stress -activated protein kinase signaling cascade |
| g35768_i0 | MAPKKK18B | Osmotic stress -activated protein kinase signaling cascade |
| g52606_i0 | MAPKKK18C | Osmotic stress -activated protein kinase signaling cascade |
| g25762_i0 | MAPKKK17A | Osmotic stress -activated protein kinase signaling cascade |
| g27685_i0 | MAPKKK17 | Osmotic stress -activated protein kinase signaling cascade |
| g14306_i0 | LRK10L1 | Receptor-like serine/threonine-protein kinase response to water deprivation |
| g22198_i0 | HK2 | Histidine kinase; Response to water deprivation |
| g41313_i0 | GCN2 | Translation initiation factor 2alpha kinase; Cellular response to hydrogen peroxide |
| g11679_i0 | RDUF2 | E3 ubiquitin-protein ligase; Response to water deprivation |
| g34536_i0 | BPM5 | Cellular response to water deprivation; Protein ubiquitination |
| g46237_i0 | BPM2 | Cellular response to water deprivation; Protein ubiquitination |
| g43372_i0 | SOD2A | Superoxide dismutase; Reactive oxygen species metabolic process |
| g49439_i0 | SOD2B | Superoxide dismutase; Reactive oxygen species metabolic process |
| g76468_i0 | SOD1A | Superoxide dismutase; Reactive oxygen species metabolic process |
| g73867_i0 | SOD1B | Superoxide dismutase; Reactive oxygen species metabolic process |
| g26922_i0 | PODc2 | Peroxidase; Hydrogen peroxide catabolic process |
| g64569_i0 | TPx | Cellular response to osmotic stress; Hydrogen peroxide catabolic process |
| g23229_i0 | POD N | Peroxidase; Hydrogen peroxide catabolic process |
| g29859_i0 | POD 2 | Peroxidase; Hydrogen peroxide catabolic process |
| g24903_i0 | POD 55 | Peroxidase; Hydrogen peroxide catabolic process |
| g32263_i0 | pmPOD 2 | Peroxidase; Hydrogen peroxide catabolic process |
| g15346_i0 | Peroxisomal CAT | Peroxisomal catalase; Hydrogen peroxide metabolic process |
| g38319_i0 | Ligninase-3 | Hydrogen peroxide catabolic; Response to oxidative stress |
| g103443_i0 | GRIM-19 | NADH dehydrogenase; Reactive oxygen species metabolic process |
| g39216_i0 | Glx I | Cellular detoxification; Cellular response to osmotic stress |
| g11107_i0 | CP | Catalase; Hydrogen peroxide catabolic process |
| g26822_i0 | CLCN3 | Chloride transporter; Regulation of reactive oxygen species metabolic process |
| g21010_i0 | CCP | Peroxidase; Hydrogen peroxide catabolic process |
| g8640_i0 | CATa | Catalase; Hydrogen peroxide catabolic process |
| g16715_i0 | CATb | Catalase; Hydrogen peroxide catabolic process |
| g20050_i0 | CATc1 | Catalase; Hydrogen peroxide catabolic process |
| g56494_i0 | Apo-D | Apolipoprotein; Response to reactive oxygen species |
| g7378_i0 | Hsp70 | ATPase activity; Cellular response to osmotic stress |
| g11152_i0 | Cpn60 | Cellular response to osmotic stress; Protein refolding |
| g14939_i0 | PP2C-2 | Negative regulation of stress-activated MAPK cascade |
| g27894_i0 | HK1 | Negative regulation of abscisic acid-activated signaling pathway; Regulation of stomatal closure |
| g66294_i0 | EXO70B1a | Positive regulation of abscisic acid-activated signaling pathway; Response to water deprivation |
| g35007_i0 | EXO70B1b | Positive regulation of abscisic acid-activated signaling pathway; Response to water deprivation |
| g3646_i0 | ELF3 | Abscisic acid-activated signaling pathway; Response to water deprivation |
| **Genes down-regulated in Sopin** | | |
| **Gene ID** | **Gene name** | **Description** |
| g13053_i0 | NAC6e | Transcription factor; Regulation of response to water deprivation |
| g44277_i0 | DREB1B2 | Dehydration-responsive element-binding protein; Transcription factor |
| g43577_i0 | ACA5a | Response to water deprivation; Integral component of plasma membrane |
| g20139_i0 | Trehalose synthase | Trehalose synthase; Glucose metabolic process |
| g19262_i0 | UGPase | UDP-glucose pyrophosphorylase; Trehalose biosynthetic process |
| g2590_i0 | CESA1 | Cellulose synthase catalytic subunit A; Carbohydrate catabolic process |
| g16485_i0 | CESA2 | Cellulose synthase catalytic subunit A; Carbohydrate catabolic process |
| g6358_i0 | TPS4 | Trehalose metabolism in response to stress |
| g43012_i0 | TPP2A | Trehalose-phosphatase; Trehalose biosynthetic process |
| g25936_i0 | TPP2B | Trehalose-phosphatase; Trehalose biosynthetic process |
| g22815_i0 | MST4 | Sugar transport protein; Response to water deprivation |
| g7366_i0 | FEH | β-fructofuranosidase activity; Carbohydrate metabolic process |
| g11296_i0 | CslA3 | Cellulose synthase-like protein A3; Carbohydrate catabolic process |
| g8000_i0 | 1-SSTb | Sucrose:sucrose 1-fructosyltransferase; Carbohydrate metabolic process |
| g8419_i0 | 1-SSTc | Sucrose:sucrose 1-fructosyltransferase; Carbohydrate metabolic process |
| g60206_i0 | RBG4 | Mitochondrial RNA-binding protein; Response to water deprivation |
| g15229_i0 | DDX3X | ATP-dependent RNA helicase; Cellular response to osmotic stress |
| g39510_i0 | RD19A | Response to osmotic stress |
| g9843_i0 | P5CS | δ-1-pyrroline-5-carboxylate synthase; L-proline biosynthetic process |
| g47915_i0 | P5CR2 | Pyrroline-5-carboxylate reductase; L-proline biosynthetic process |
| g33594_i0 | PLP | Proline synthase co-transcribed homolog protein |
| g24683_i0 | TPP3 | Trehalose-phosphatase; Trehalose biosynthetic process |
| g46829_i0 | PP2Cb | Negative regulation of stress-activated MAPK cascade |
| g80274_i0 | SAPK8 | Osmotic stress -activated protein kinase signaling pathway |
| g32663_i0 | MAPKKK17C | Osmotic stress -activated protein kinase signaling cascade |
| g45741_i0 | FERLP1 | Cellular response to osmotic stress; Golgi apparatus |
| g55901_i0 | YbgG | Betaine-homocysteine S-methyltransferase |
| g48254_i0 | BADHa | Betaine-aldehyde dehydrogenase; Glycine betaine biosynthetic process |
| g21190_i0 | BADHb | Betaine-aldehyde dehydrogenase; Glycine betaine biosynthesis |
| g22109_i0 | CAP1 | Adenylyl cyclase-associated protein; Response to osmotic stress |
| g50315_i0 | BPM2C | Cellular response to water deprivation; Protein ubiquitination |
| g27511_i0 | BPM2B | Cellular response to water deprivation; Protein ubiquitination |
| g23272_i0 | BPM1A | Cellular response to water deprivation; Protein ubiquitination |
| g33323_i0 | BPM1 | Cellular response to water deprivation; Protein ubiquitination |
| g13030_i0 | TR1 | Thioredoxin reductase; Hydrogen peroxide catabolic process |
| g24173_i0 | TPO | Peroxidase; Hydrogen peroxide catabolic process |
| g1810_i0 | Spastin | Reactive oxygen species metabolic process |
| g46512_i0 | Sestrin1 | Peroxiredoxin; Cellular oxidant detoxification |
| g45171_i0 | POD2B | Peroxidase; Hydrogen peroxide catabolic proces |
| g50354_i0 | POD2C | Thioredoxin peroxidase; Hydrogen peroxide catabolic process |
| g68774_i0 | POD2D | Thioredoxin peroxidase; Hydrogen peroxide catabolic process |
| g33416_i0 | PODA | Thioredoxin peroxidase; Hydrogen peroxide catabolic process |
| g15011_i0 | NOX-1 | NADPH oxidase; Positive regulation of reactive oxygen species metabolic process |
| g16927_i0 | CATd | Catalase; Hydrogen peroxide catabolic process |
| g38434_i0 | CATc2 | Catalase; Hydrogen peroxide catabolic process |
| g10455_i0 | CAT isozyme | Catalase; Hydrogen peroxide catabolic process |
| g33741_i0 | CATe | Catalase; Hydrogen peroxide catabolic process |
| g19492_i0 | ACA5b | Calcium-transporting ATPase 5; Response to water deprivation |
| g50251_i0 | CBL | Calcium ion binding; Abscisic acid-activated signaling pathway |
