# Supplementary material for: The genetic variation in drought resistance in eighteen perennial ryegrass varieties and the underlying adaptation mechanisms
Source: BMC Plant Biol. 2023 Sep 26;23:451. doi: 10.1186/s12870-023-04460-z (PMC10521523; doi:10.1186/s12870-023-04460-z)
Supplement: Supplementary file 5 — Supplementary Material 5 [file 12870_2023_4460_MOESM5_ESM.docx]

| **Table S4. Primers used in RT-qPCR in this study** | | | |
| --- | --- | --- | --- |
| **Gene ID** | **Gene name** | **Primer name** | **Sequence** |
| g18214_i0 | AMY6 | AMY6-5’ | CCGGTGAGAAGTGGACTGAC |
|  |  | AMY6-3’ | GCATTCTACAGCAAACCGCC |
| g11438_i0 | AMY7 | AMY7-5’ | AGTTTCCAGCTCATCGGTGG |
|  |  | AMY7-3’ | TTTACTGCTACGGCGACCTG |
| g44115_i0 | MGAM | MGAM-5’ | CTTCGCTCGCGGGATCTATT |
|  |  | MGAM-3’ | GTCGTATAGACCGGTGGCAG |
| g14513_i0 | PFK3 | PFK3-5’ | ATGACCATGTGGCCGTTGTC |
|  |  | PFK3-3’ | TCGACAAGTCCTTCGGCTTC |
| g15007_i0 | GPI | GPI-5’ | GCGCCATCTGGAACATCAAC |
|  |  | GPI-3’ | CAGCCATTACAGCCTGGTCA |
| g23432_i0 | treT | treT-5’ | GGCAAAACGCTCTCATCGTC |
|  |  | treT-3’ | AGGTAGGGTCCTCGACCATC |
| g8839_i0 | GBE1 | GBE1-5’ | TCTGAGCTTTGGAGAAGCGG |
|  |  | GBE1-3’ | TGTGAAACCTGCATCCTCCC |
| g34417_i0 | TPS1 | TPS1-5’ | TAGTCGAGGCGAACAACACC |
|  |  | TPS1-3’ | GGAGTCGAGTTCAAGGGCAA |
| g24695_i0 | TPP7A | TPP7A-5’ | ATAATGATGGCGCGTGCTTG |
|  |  | TPP7A-3’ | CTTGCCCTTGTCCCACTTGA |
| g20216_i0 | Hexokinase | Hexokinase-5’ | TGGTGTTGGTATACGCGGAG |
|  |  | Hexokinase-3’ | CCTCTCGGCTTCACCTTCTC |
| g11296_i0 | CslA3 | CslA3-5’ | GGAGCATGGTGGTTTCCTGA |
|  |  | CslA3-3’ | GGCGACGATATGCCTTCAGA |
| g73867_i0 | SOD1A | SOD1A-5’ | TGGAAACATCGAGACGGACG |
|  |  | SOD1A-3’ | GAGATCATCCGTGCCAGCAT |
| g73867_i0 | SOD1B | SOD1B-5’ | CACCATTGTCGTTCACGCTG |
|  |  | SOD1B-3’ | GCCAATAACACCGCAAGCAG |
| g49439_i0 | SOD2B | SOD2B-5’ | GCGATTTGCTCGCTGAAGTT |
|  |  | SOD2B-3’ | CCCGACCTCCCATACGACTA |
| g8640_i0 | CATa | CATa-5’ | GCCCCAAGCACAACAAGAAG |
|  |  | CATa-3’ | GCCTTGATGCCGTTGTAAGC |
| g15346_i0 | Peroxisomal CAT | Peroxisomal CAT-5’ | GCTCCTTGAAGCTGATGGGT |
|  |  | Peroxisomal CAT-3’ | GCGCCAACTACCAACAACTG |
| g26922_i0 | PODc2 | PODc2-5’ | GGGCTTCAGCGTAATCGACA |
|  |  | PODc2-3’ | GTTCTTCTTGGAGAACGCGG |
| g32263_i0 | pmPOD2 | pmPOD2-5’ | CGCCAAGCAAAGTATTCGGG |
|  |  | pmPOD2-3’ | ATATCTCCACCGAAAGGCGG |
| g11107_i0 | CP | CP-5’ | CTCCTCGACAACAACACCGA |
|  |  | CP-3’ | TGTGCACTCACCGACTTGTT |
| g21010_i0 | CCP | CCP-5’ | GGCGATGTGGTTGTAGACCT |
|  |  | CCP-3’ | CGCTTCTCAGGAGGATGGAC |
| g26822_i0 | CLCN3 | CLCN3-5’ | GTGAGCCCCAAGACGAAGAA |
|  |  | CLCN3-3’ | AGACCAGCGTACTTGCCATC |
| g45171_i0 | POD2B | POD2B-5' | GGCAGCCGCGAAGTCCTGCCT |
|  |  | POD2B-3' | CCACAGGGCAGGCGCAGTGT C |
| g50354_i0 | POD2C | POD2C-5' | CGTCCCTGGGGCCTTCACCC |
|  |  | POD2C-3' | CCCAAACCTTTCTCTGAAAG |
| g43700_i0 | WRKY30 | WRKY30-5’ | TCGGTCACATAAGCTGTGGC |
|  |  | WRKY30-3’ | CAGCGCACAGACAATTACCC |
| g48499_i0 | WRKY54 | WRKY54-5’ | GTCGTGGAAGAAGAGGCGTA |
|  |  | WRKY54-3’ | CGAATCCCAACCTGCCCTAT |
| g32480_i0 | NAC6B | NAC6B-5’ | CTCCCACTCGGTGATCTTGG |
|  |  | NAC6B-3’ | CGTGCTAACGGGGATGATGA |
| g50496_i0 | NAC6D | NAC6D-5’ | GCTCCCACTCGGTGATCTTG |
|  |  | NAC6D-3’ | CGGGGATGATGAACCAGACC |
| g43301_i0 | NAC22 | NAC22-5’ | TCAGACGCGGATCTCACAAC |
|  |  | NAC22-3’ | AGACGGTGAAAGTAGGCACC |
| g41924_i0 | DREB1B | DREB1B-5’ | GTCGTCGCGTTCCAGCTAT |
|  |  | DREB1B-3’ | GCGCAAGGCTCTCGTAGTA |
| g27234_i0 | DREB1C | DREB1C-5’ | GAGCTACTGATCGACCCAGC |
|  |  | DREB1C-3’ | GCTCCAGACCAACTCTGCTT |
| g38459_i0 | DREB1H | DREB1H-5’ | GCTCGCAGAAAGAACTTCGC |
|  |  | DREB1H-3’ | GTACTACATGAGCCTGGCGG |
| g26761_i0 | MAPKKK18A | MAPKKK18A-5’ | GACTGGGACTCGAACGAAGG |
|  |  | MAPKKK18A-3’ | CCGGAACATACTCGTCCTCG |
| g35768_i0 | MAPKKK18B | MAPKKK18B-5’ | AGATGTCAAGGGCAAGTGGG |
|  |  | MAPKKK18B-3’ | CGACTACCTGTGCGTCAGTT |
| g22198_i0 | HK2 | HK2-5’ | AGCTCCTTGGGTATGAGGGT |
|  |  | HK2-3’ | TTGACGGAGTTCGCATCCAA |
| g6992_i0 | PBS2 | PBS2-5’ | CCCAAACTCCAGATGTCGCT |
|  |  | PBS2-3’ | ATGAACACCAAGGGTGCGAT |
| g46237_i0 | BPM2 | BPM2-5’ | TAGTGCCAAGATGGTCGCTG |
|  |  | BPM2-3’ | GCTCATTGCCAGAAACGGTG |
| g11679_i0 | RDUF2 | RDUF2-5’ | GCTGTAGCACCAGTAGGAGG |
|  |  | RDUF2-3’ | AGAAAGCTCCCTTCTTGCGG |
| g3646_i0 | ELF3-5 | ELF3-5’ | GAACGACACGCTCATCAAGC |
|  | ELF3-3 | ELF3-3’ | GGCCGTACCCAAAGAAAACG |
| g17091_i0 | GPT | GPT-5’ | TCAAGAAGGCTCTACGCGAC |
|  |  | GPT-3’ | GTGGAAGATGCCGTCGTACT |
| g4997_i0 | AGK | AGK-5’ | CGTCTTCTCCAGGTGCCATT |
|  |  | AGK-3’ | ACGCGACCATTTGAGGTCTT |
| g23596_i0 | ACOAT | ACOAT-5’ | TTAACCGCGTCCACATCGTT |
|  |  | ACOAT-3’ | AGCGCCACTCGTACATTCAT |
| g24814_i0 | OAT2 | OAT2-5’ | CCGTCTCCGTTGTCCTTACC |
|  |  | OAT2-3’ | TCCCTTGCCTCCTCTGACTT |
| g24110_i0 | P5CR1 | P5CR1-5’ | TGGTCGCCATGGTGGATATG |
|  |  | P5CR1-3’ | GCCACCTGCATAGTGTGTCA |
| g9843_i0 | P5CS | P5CS-5’ | ACGATCGGAATGACGTCGAG |
|  |  | P5CS-3’ | GCGCCTCAACAAGCAGATTC |
| g6773_i0 | 1-SSTa | 1-SSTa-5’ | GTACGCCGCCGTCTGTTCG |
|  |  | 1-SSTa-3’ | CTTCTACGACCCGACCAAGAAC |
| g8000_i0 | 1-SSTb | 1-SSTb-5’ | GCCTCGATGTCGAGCTGCATGG |
|  |  | 1-SSTb-3’ | CTGGGGAAAGTTCTACGCATC |
| g8419_i0 | 1-SSTc | 1-SSTc-5’ | CCGTTTCTTGGCCGGGTCGTAG |
|  |  | 1-SSTc-3’ | CCGCCTGGTTCGACGAGTCC |
| g7366_i0 | FEH | FEH -5’ | GCTCCTGCAGATCGCCCGAG |
|  |  | FEH -3’ | CGACACCGAGGCCGACTACGTC |
|  | TEF1 | TEF1-5’ | CGTGTGATCGAGAGGTTTGA |
|  |  | TEF1-3’ | CGAATTTCCAGAGGGCAATA |
|  | eEF1A | eEF1A -5’ | CCGTTTTGTCGAGTTTGGT |
|  |  | eEF1A -3’ | AGCAACTGTAACCGAACATAGC |
